# Characteristics and immune checkpoint status of radioiodine-refractory recurrent papillary thyroid carcinomas from Ukrainian Chornobyl Tissue Bank donors

**DOI:** 10.3389/fendo.2023.1343848

**Published:** 2024-01-08

**Authors:** Tetiana Bogdanova, Tatiana I. Rogounovitch, Liudmyla Zurnadzhy, Norisato Mitsutake, Mykola Tronko, Masahiro Ito, Michael Bolgov, Serhii Chernyshov, Serhii Gulevatyi, Sergii Masiuk, Shunichi Yamashita, Vladimir A. Saenko

**Affiliations:** ^1^ Laboratory of Morphology of Endocrine System, State Institution “VP Komisarenko Institute of Endocrinology and Metabolism of the National Academy of Medical Sciences of Ukraine”, Kyiv, Ukraine; ^2^ Department of Radiation Molecular Epidemiology, Atomic Bomb Disease Institute, Nagasaki University, Nagasaki, Japan; ^3^ Department of Radiation Medical Sciences, Atomic Bomb Disease Institute, Nagasaki University, Nagasaki, Japan; ^4^ Department of Fundamental and Applied Problems of Endocrinology, State Institution “VP Komisarenko Institute of Endocrinology and Metabolism of the National Academy of Medical Sciences of Ukraine”, Kyiv, Ukraine; ^5^ Department of Diagnostic Pathology, National Hospital Organization Nagasaki Medical Center, Omura, Japan; ^6^ Department of Surgery of Endocrine Glands, State Institution “VP Komisarenko Institute of Endocrinology and Metabolism of the National Academy of Medical Sciences of Ukraine”, Kyiv, Ukraine; ^7^ Laboratory of Radiology and Radiobiology, State Institution “VP Komisarenko Institute of Endocrinology and Metabolism of the National Academy of Medical Sciences of Ukraine”, Kyiv, Ukraine; ^8^ Radiation Protection Laboratory, State Institution “National Research Center of Radiation Medicine of the National Academy of Medical Science of Ukraine”, Kyiv, Ukraine; ^9^ Global Exchange Center, Fukushima Medical University, Fukushima, Japan

**Keywords:** radioiodine-refractory recurrent papillary thyroid carcinoma, Chornobyl tissue bank, radiation exposure, pathology, immune checkpoint status, PD-L1, PD-1, p16 INK4a

## Abstract

**Introduction:**

The radioiodine-refractory (RAI-R) recurrent papillary thyroid carcinomas (PTCs) are more frequent in elderly patients and have an unfavorable prognosis. Data on the prevalence and characteristics of RAI-R recurrent PTCs in patients of young and middle age with or without a history of radiation exposure in childhood are poorly described. The aim of the current study was: i) to determine the frequency of RAI-R recurrent PTCs among donors of the Chornobyl Tissue Bank (CTB) and analyze the clinicopathological features of primary tumors (PTs), primary metastases (PMTSs), recurrent metastases (RMTSs) and risk factors for RMTS, and ii) to determine the immune checkpoint status (ICS) of the RAI-R recurrent PTCs and to assess the factors associated with ICS positivity.

**Methods:**

Sixty RAI-R recurrent PTCs (46 exposed to radiation and 14 non-exposed, 2.5% of all cases registered with the CTB) from the Ukrainian patients aged up to 48 years were identified.

**Results:**

The clinicopathological characteristics of the PTs moderately to weakly resembled those of the PMTS and RMTS from the same patients while the metastatic tissues were highly similar. The multivariate model of RMTS included the dominant solid-trabecular growth pattern of the PT, cystic changes, N1b metastases, and the probability of a causation (POC) of PTC by radiation as risk factors. Among these factors, the lateral PMTS (N1b) had the strongest effect. The longer period of latency (a POC component) was the second statistically significant characteristic. ICS percent agreement between the PT and RAI-R RMTS was 91.5%; 23.7% of PTs and 28.8% of RMTSs had positive ICS (positive PD-L1 tumor epithelial cells (TECs) and positive PD-L1/PD1 tumor-associated immune cells). ICS positivity of PTs was associated with pronounced oncocytic changes and high density of the p16^INK4A^-positive TECs in the invasive areas of PTs. In RMTSs, ICS positivity was associated with pronounced oncocytic changes and Ki-67 labeling index ≥ 4.5% of PTs, and the dominant solid-trabecular growth pattern, Ki-67 labeling index ≥ 7.6% and p16^INK4A^-positivity of RMTS.

**Discussion:**

The findings are of clinical relevance and may be useful for developing individual treatment approaches for patients with RAI-R recurrent PTCs possibly involving immunotherapy.

## Introduction

Papillary thyroid carcinoma (PTC) accounts for more than 90% of differentiated thyroid cancer cases (1–3). Depending on the initial risk stratification, treatment of PTC may include thyroid surgery of various extent, radioiodine (RAI) therapy, and hormone suppression/replacement therapy resulting generally in a very good prognosis with a 5-year relative patient survival of 94-98% (2), and a > 90% 10-year overall survival (4–7). However, 5-20% of patients may experience a local, regional or distant recurrence (2, 4). Furthermore, 5-15% of differentiated thyroid carcinomas may be insensitive to RAI therapy (8–10). The RAI-refractory (RAI-R) tumors may be a life-threatening condition reducing a 5-year disease-specific survival rate to 60-70% (11, 12), and a 10-year survival rate to as low as 10-20% for patients with RAI-R metastatic malignancies (6, 13–16).

The RAI-R recurrent PTCs occur more often in elderly patients who have the most unfavorable prognosis (16–19). Young and middle-aged patients (< 45 years old) have a better overall survival (19), but data on the frequency of RAI-R recurrent PTC in patients of this age with a history of radiation exposure in childhood, and clinicopathological characteristics of primary tumors, primary metastases and recurrent metastases are poorly described. In this regard, the international Chornobyl Tissue Bank (CTB), which consists of > 80% tumor samples from the Ukrainian donors with demographic, clinical and histopathological data, individual radiation thyroid doses and a consensus CTB diagnosis, provides a unique opportunity to address these questions (20, 21).

In view of unfavorable prognosis, management of RAI-R recurrent PTC is a difficult and important clinical problem. Current modalities may include surgery, external beam radiotherapy and targeted multi-kinase inhibitor treatment (22). In recent years, the immune checkpoint inhibitor therapy started to gain increasing attention (23–27). Possible effectiveness of the immunotherapy of RAI-R recurrent PTC was hypothesized because the microenvironment of differentiated thyroid cancer is enriched with different types of immune cells (28). However, some tumors can evade the immune response by expressing the programmed cell death ligand PD-L1, which binds the PD-1 receptor on T-lymphocytes and disrupts cytotoxic activity of the latter, leading to higher tumor aggressiveness and progression (23, 25, 28). The PD-1/PD-L1 immune checkpoint is the principal target of immunotherapy for various malignant tumors, including the RAI-R recurrent PTC (24, 27, 29, 30). Again, our knowledge of the immune checkpoint status of the RAI-R recurrent PTCs in young and middle-aged patients exposed or non-exposed to radiation is insufficient.

This study set out i) to determine the frequency of RAI-R recurrent PTCs among the Chornobyl Tissue Bank (CTB) donors, to analyze clinicopathological characteristics of the primary tumors (PT), primary metastases (PMTS) and recurrent metastases (RMTS), to ascertain risk factors for RMTS development, and ii) to determine the frequency of a positive immune checkpoint status (ICS) among the RAI-R recurrent PTCs and to identify the clinicopathological characteristics of PT, PMTS and RAI-R RMTS associated with positive ICS.

## Materials and methods

### Patients

Clinical records and follow-up data on 3,595 Ukrainian patients operated on for benign or malignant thyroid tumors at the State Institution “V.P. Komisarenko Institute of Endocrinology and Metabolism of the National Academy of Medical Sciences of Ukraine” (IEM, Kyiv, Ukraine) during the period from 1998 to 2017 were reviewed. A total of 2,411 patients with PTCs, including micro-PTCs, all registered with the CTB, were identified, among whom 60 patients were re-operated for the RAI-R recurrences. Recurrence was defined as a regional metastasis newly detected not earlier than six months after the initial treatment. Follow-up period ranged from 1 to 21 years, median 8.4; no fatal outcomes were documented. The RAI-R status was determined according to the existing guidelines (4, 5, 7). All patients underwent two-projection whole-body scintigraphy (RAI-activity from 2,000 to 5,500 MBq) at different times after the first surgery (**Table 1**) and displayed no abnormal RAI uptake. On ultrasound, however, enlarged hypoechoic lymph nodes measuring from 7 to 15 mm were detected in the neck, which according to fine-needle aspiration biopsy/cytology and postoperative histopathology were classified as recurrent PTC metastases. Formalin-fixed paraffin-embedded tissues samples of the primary tumor, primary (where existed) and recurrent metastases were retrieved from the pathological archive and analyzed as described below.

**Table 1:** Descriptive characteristics of the RAI-R recurrent PTC cases: primary tumors, primary metastases and recurrent RAI-R metastases.

Parameters	Primary tumors, n=60	Primary LN metastases, n=39	Recurrent metastases, n=60
	n (%) or median (range; IQR)	n (%) or median (range; IQR)	n (%) or median (range; IQR)
**Exposed/nonexposed** (% exposed)	46/14 (76.7%)	27/12 (69.2%)	PT^1^
**Radiation dose to the thyroid**, mGy	n=46; 32.1 (2.3-825.1; 20.9-64.4)	n=27; 34.7 (3.1-801.6; 21.1-88.5)	NA^2^
**Probability of causation (POC),** %	n=46; 12.4 (0.8-86.5; 6.5-31.2)	n=27; 13.5 (0.8-79.5; 8.2-39.9)	NA
≤ 25%	32 (69.6%)	16 (59.3%)	NA
> 25 – 50%	6 (13.0%)	6 (22.2%)	NA
> 50 – 75%	6 (13.0%)	4 (14.8%)	NA
> 75 – 100%	2 (4.4%)	1 (3.7%)	NA
**Age at operation**, years	28.4 (8.7-48.4; 22.5-35.5)	27.6 (8.7-46.4; 21.8-35.1)	32.6 (9.9-49.0; 24.8-37.9)
**Age at exposure**, years	n=46; 9.6 (0-18.3; 4.2-13.0)	n=27; 9.3 (0-18.1; 4.0-13.1)	PT
**Period of latency**, years	n=46; 22.9 (12.6-31.0; 19.4-26.9)	n=27; 23.9 (12.6-30.4; 21.6-27.2)	NA
**Sex F/M** (%M, F:M ratio)	44/16 (26.7%; 2.7:1)	31/8 (20.5%; 3.9:1)	PT
**Tumor size**, mm	20.0 (6-105; 14-35)	16 (3-100; 9-32)	13.0 (6-25; 10-15)
≤ 10 mm (microcarcinoma)	7 (11.7%)	NA	NA
11 – 20 mm	25 (41.7%)	NA	NA
21 – 40 mm	20 (33.3%)	NA	NA
> 40 mm	8 (13.3%)	NA	NA
**Dominant growth pattern**			
papillary	40 (66.7%)	18 (46.2%)	29 (48.3%)
follicular	5 (8.3%)	5 (12.8%)	4 (6.7%)
solid-trabecular	15 (25.0%)	16 (41.0%)	27 (45.0%)
**Histological subtype**			
papillary	14 (23.3%)	9 (23.1%)	16 (26.7%)
follicular	2 (3.3%)	3 (7.7%)	2 (3.3%)
solid-trabecular	5 (8.3%)	12 (30.8%)	23 (38.3%)
conventional	29 (48.3%)	11 (28.2%)	15 (25.0%)
rare	10 (16.7%)^3^	4 (10.3%)^4^	4 (6.7%)^5^
**Tall cell features**	27 (45.0%)	14 (35.9%)	26 (43.3%)
**Hobnail features**	4 (6.7%)	3 (7.7%)	10 (16.7%)
**Full tumor capsule**	4 (6.7%)	NA	NA
**Multifocality**	19 (31.7%)	NA	NA
**Lymphatic/vascular invasion**	45 (75.0%)	NA	NA
**Extrathyroidal extension** (any)	35 (58.3%)	13 (33.3%)	23 (38.3%)
microscopic	26 (43.3%)	NA	NA
macroscopic	9 (15.0%)	NA	NA
**Extranodal extension**	NA	13 (33.3%)	23 (38.3%)
**pT category**			
pT1	32 (53.3%)	NA	NA
pT1a	7 (11.7%)	NA	NA
pT1b	25 (41.6%)	NA	NA
pT2	12 (20.0%)	NA	NA
pT3	16 (26.7%)	NA	NA
pT3a	4 (6.7%)	NA	NA
pT3b	12 (20.0%)	NA	NA
**pN category** (N1)	39 (65.0%)	NA	NA
pN1a	15 (25.0%)	NA	NA
pN1b	24 (40.0%)	NA	NA
**M category** (M1)	1 (1.7%)	NA	NA
**Oncocytic changes**	49 (81.7%)	31 (79.5%)	48 (80.0%)
≤ 25% focal	9 (15.0%)	7 (17.9%)	7 (11.7%)
> 25 – 50% moderate	24 (40.0%)	10 (25.7%)	16 (26.7%)
> 50 – 75% severe	13 (21.7%)	9 (23.1%)	11 (18.3%)
> 75 – 100% oncocytic tumor	3 (5.0%)	5 (12.8%)	14 (28.3%)
**Cystic changes**	15 (25.0%)	14 (35.9%)	29 (48.3%)
≤ 25% focal	14 (23.3%)	8 (20.5%)	9 (15.0%)
> 25 – 50% moderate	1 (1.7%)	3 (7.7%)	10 (16.7%)
> 50 – 75% severe	0	1 (2.6%)	2 (3.3%)
> 75 – 100% cystic tumor	0	2 (5.1%)	8 (13.3%)
**BRAF^V600E^-positive**	n=59; 41 (69.5%)	n=35; 24 (68.6%)	n=59; 41 (69.5%)
**NRAS^Q61R^-positive**	n=59; 1 (1.7%)	n=35; 1 (2.9%)	1 (1.7%)
**Ki-67 labeling index**, %	n=59; 5.1 (1.0-14.3; 3.7-7.3)	n=34; 4.6 (1.2-12.2; 3.3-7.1)	n=59; 5.4 (1.6-14.6; 3.5-7.3)
0 – 5%	29 (49.2%)	18 (52.9%)	27 (45.8%)
> 5 – 10%	24 (40.7%)	13 (38.2%)	30 (50.8%)
> 10%	6 (10.1%)	3 (8.9%)	2 (3.4%)
**p16-positive TEC^6^, invasive areas**	n=59; 58 (98.3%)	n=35; 33 (94.3%)	n=59; 59 (100%)
≤ 25%	18 (30.5%)	7 (20.0%)	12 (20.3%)
> 25 – 50%	14 (23.7%)	7 (20.0%)	12 (20.3%)
> 50 – 75%	20 (33.9%)	7 (20.0%)	16 (27.1%)
> 75 – 100%	6 (10.2%)	12 (34.3%)	19 (32.3%)
**Coexisting thyroid cancer**	0	NA	NA
**Concomitant benign nodules**	14 (23.3%)	NA	NA
**Concomitant Graves’ disease**	1 (1.7%)	NA	NA
**Chronic thyroiditis**	15 (25.0%)	NA	NA
**Total thyroidectomy**	60 (100%)	NA	NA
**Lymph node dissection performed**	47 (78.3%)	39 (100%)	60 (100%)
level ≥ 6	16 (26.6%)	12 (30.8%)	27 (45.0%)
level 1 – 5	31 (51.7%)	27 (69.2%)	33 (55.0%)
**Lymph nodes removed**	NA	10 (1-29; 5-14)	4.5 (1-22; 2-6)
**Metastatic lymph nodes**	NA	7 (1-17; 3-10)	2.5 (1-18; 1-4)
**Greatest metastatic lymph node size,** mm	NA	16 (3-100; 9-33)	13 (6-25; 10-15)
**RIT performed**	60 (100%)	NA	NA
**RIT cycles**	2 (1-10; 1-2)	NA	NA
**Cumulative RI activity**, MBq	5455 (1425-54036; 3787-8338)	NA	NA
**Follow-up**, years	8.4 (1-21; 4.8-13.9)	NA	NA
**Time to recurrence**, yrs	NA	NA	1.6 (0.5-19.5; 0.9-3.9)

^1^ Identical to the primary tumor data.

^2^ Not applicable.

^3^ Seven tall cell and three Warthin-like subtypes.

^4^ Three tall cell and one hobnail subtypes.

^5^ Four tall cell subtypes.

^6^ Tumor epithelial cells.

The study was conducted according to the guidelines of the Declaration of Helsinki and was approved by the IEM Bioethics Committee (protocols N 22-KE of April 26, 2018, and N 31-KE of February 27, 2020), the Chornobyl Tissue Bank (CTB, project N001-2020), and the Ethics Committee of Nagasaki University (protocol 20130401–7 of July 1, 2021, the latest update). Informed consent was obtained from all patients enrolled in the study or their guardians (for minors).

### Histopathology

Pathological examination of paraffin sections stained with hematoxylin and eosin was performed by two experienced IEM pathologists (TB and LZ). Pathological diagnosis was based on the 4th edition of the WHO histological classification (31). All tumors had been also reviewed by the international pathology panel of the CTB project (32), and PTC diagnosis was confirmed in all cases. pTNM categories were determined according to the 8th edition of the TNM Classification (33). Tumors were classified by size, the presence of a capsule, dominant histological growth pattern (papillary, follicular or solid-trabecular) and histological subtype. The presence of tall cell and of hobnail areas, and the frequency of oncocytic changes were recorded. In case of multifocal PTs or multiple primary or recurrent lymph node involvement, characteristics of the largest were considered.

### Immunohistochemistry

Immunohistochemical (IHC) staining for PD-L1, PD-1, p16^INK4A^ and BRAF^V600E^ were performed according to the Department of Radiation Molecular Epidemiology of the Atomic Bomb Diseases Institute’s (Nagasaki, Japan) laboratory-developed tests (TIR).

#### PD-L1, PD-1 and p16

Heat-induced epitope retrieval was performed for 15 min in VENTANA Cell Conditioning Solution (CC1) (950-124, Roche Diagnostics, Mannheim, Germany) at 120°C followed by slow cooling down during 2 h after a closed autoclave has reached 60°C upon the heating cycle completion. Endogenous peroxidase neutralization was done for 5 min with Leica Peroxidase Block (component of the Novolink Polymer Detection System (250T), RE7140-K, Leica Biosystems); non-specific blocking was performed for 5 min with Leica Protein Block (component of the Novolink Polymer Detection System (250T), RE7140-K, Leica Biosystems).

Incubation with the primary antibody: prediluted VENTANA PD-L1 (SP263) (790-4905, Roche Diagnostics) rabbit monoclonal antibody (~ 1.61 µg/ml) for 1 h at 37°C; prediluted CELL MARQUE PD-1 (NAT105) (760-4895, Roche Diagnostics) mouse monoclonal antibody (4 µg/ml) for 20 min at 37°C; prediluted VENTANA CINtec p16 Histology (705-4713, Roche Diagnostics) mouse monoclonal antibody (~ 1.0 µg/ml) for 15 min at 37°C in a wet chamber.

The Novolink Polymer Detection System (250T) (RE7140-K, Leica Biosystems) was used to detect the IHC reaction product, which included 30 min treatment with the secondary rabbit anti-mouse IgG antibody (for PD-1 and p16, omitted for PD-L1), attachment of the Novolink Polymer for 30 min and visualization with DAB diluted in the Novolink DAB Substrate Buffer according to the manufacturer’s recommendations. Cell nuclei were stained with Mayer’s hematoxylin. Placenta and tonsil tissue sections were used as positive controls for PD-L1; tonsil tissue sections were used as positive controls for PD-1 and p16 staining.

PD-L1 expression in tumor epithelial cells (TECs) was determined as the percentage of TЕCs with membrane and cytoplasmic staining. PD-L1 expression in tumor-associated immune cells (TAIC) was determined as the percentage of TAIC with membrane and cytoplasmic staining. PD-1 expression was determined as the percentage of TAICs with cytoplasmic staining. p16^INK4A^ expression was determined in the tumor invasive areas in TECs with nuclear and cytoplasmic staining. PD-L1 expression in TEC and TAIC, PD-1 expression in TAIC and p16^INK4A^ expression in TEC were scored as: 0:0; 1: ≤ 25%; 2: > 25% – ≤ 50%; 3: > 50% – ≤ 75%; 4: > 75% –100%.

For analysis, the immune checkpoint status (ICS) of each individual PT, PMTS or RMTS was evaluated as positive if PD-L1 expression was observed in > 25% of the TECs (i.e., score 2 or higher), and that of PD-L1 and PD-1 in any percentage of the TAICs above 0 (i.e., score > 0). The high density of PD-L1 and PD-1 positive TAICs in the germinal centers of lymph nodes with PTC metastases was not considered.

#### NRAS (mutated p.Q61R)

Heat-induced epitope retrieval was performed for 15 min in BOND Epitope Retrieval Solution 2 (AR9640, Leica Biosystems) at 120°C following by cool down to 60°C in a closed autoclave (without further incubation). Endogenous peroxidase neutralization was done for 5 min with Leica Peroxidase Block (component of the Novolink Polymer Detection System (250T), RE7140-K, Leica Biosystems); non-specific blocking was performed for 5 min with Leica Protein Block (component of the Novolink Polymer Detection System (250T), RE7140-K, Leica Biosystems).

Incubation with the Anti-NRAS (mutated Q61R) rabbit monoclonal antibody (SP174) (ab227658, Abcam) at a 1:25 dilution in freshly prepared 1% BSA in PBS was performed for 45 min at 37°C in a wet chamber.

The Novolink Polymer Detection System (250T) (RE7140-K, Leica Biosystems) was used to detect the IHC reaction product according to the manufacturer’s recommendations. Cell nuclei were stained with Mayer’s hematoxylin. Sections of a formalin-fixed paraffin-embedded PTC tissue from a patient not related to this study with the confirmed by Sanger sequencing *NRAS* c.182A>G mutation (resulting in the p.Q61R substitution) were used as a positive control. The IHC reaction was considered positive (expression of the NRAS^Q61R^ mutant protein) when a membrane and cytoplasmic staining of TECs was observed.

#### BRAF^V600E^

IHC using the anti-BRAF (mutated V600E) antibody (VE1) (ab228461, Abcam, Tokyo, Japan) was performed as described before (34). Sections of a formalin-fixed paraffin-embedded tumor tissue from a patient not related to this study with the confirmed by Sanger sequencing *BRAF^V600E^* mutation-positive PTC (35) were used as a positive control.

#### Ki-67 labeling index

The proliferative activity of TECs was evaluated by IHC using Ki-67 antibody (clone MIB-1; DAKO, Glostrup, Denmark, 1:100 dilution) in a Ventana BenchMark ULTRA instrument. Stained slides were digitally scanned with a NanoZoomer-XR (Hamamatsu, Japan) device and visualized using the NDP.view 2 software (Hamamatsu). The Ki-67 labeling index (Ki-67 LI) was determined with the image-analyzing software (CountσCell, Ki-67 antigen Semi-Auto Counter, Seiko Tec LTD, Fukuoka, Japan) in ~1,000 cells per case (TB, LZ). Image analysis was performed in a blind for the PD-L1, PD-1, p16^INK4A^, BRAF^V600E^ or NRAS^Q61R^ status manner.

### Thyroid dosimetry

^131^І thyroid radiation doses (the absorbed doses in mGy) were calculated for each patient in the Radiation Protection Laboratory of the State Institution “National Research Center for Radiation Medicine of the National Academy of Medical Sciences of Ukraine”, Kyiv using an ecological dosimetry model, which includes the system of ecological iodine transport and biokinetic models of iodine (“TD-CTB”) (36).

### Probability of causation due to radiation

The probability of causation (POC) of a tumor by exposure to a known dose of radiation of a certain quality of a subject of a given sex and age after a definite period of latency was determined using the US NIH/NCI Division of Cancer Epidemiology and Genetics’ Interactive RadioEpidemiological Program - Probability of Cancer Causation from Radiation Version 5.7.1 software [https://radiationcalculators.cancer.gov/irep, (37)] as described in our previous works (38, 39). In this study, the assigned share associated with the expected value of the excess relative risk was used as a POC estimate. The higher POC value reflects the higher likelihood of cancer development due to radiation exposure.

### Statistical analysis

Univariate analyses were performed using Fisher’s exact test for categorical data, and the Mann–Whitney test for continuous data comparison between two groups. Different coefficients of agreement and of correlation were calculated, and correspondence analysis was performed to assess the resemblance of various characteristics of the PTs, PMTSs and RMTSs (statistical tests are indicated in corresponding table footnotes or figure legends). The conditional logistic regression to analyze associations or stratified Cox proportional hazard models to analyze recurrences were run. Optimized models were created by non-automatic selection of variables using the minimization of the Akaike information criterion method. Models with small numbers of outcomes (< 5) or those with a quasi-complete separation of data points were conducted using Firth’s approach to bias-reducing penalized maximum likelihood fit or exact logistic regression. Calculations were performed using SAS 9.4 (SAS Institute, Cary, NC, USA), IBM SPSS Statistics Version 24 software (International Business Machines Corp., Armonk, NY, USA) or R (R Core Team). All tests were two-sided; p < 0.05 was considered indicative of statistical significance.

## Results

### Clinicopathological characteristics

#### Subjects and groups

From 1998 to 2017, a total of 2,411 PTC tissues from the Ukrainian patients were collected and registered with the CTB. Among those, 2,018 PTCs were from patients aged from 18.0 to 48.4 years (32.7 (25.4-36.5) years, median and IQR) who were less than 18 [9.6 (4.2-13.0)] years at the time of the Chornobyl accident. These subjects belong to the high-risk group for developing radiogenic thyroid cancer as a result of internal exposure to ^131^I (40–42). In addition, 393 sporadic PTCs were removed from patients aged from 8.7 to 25.3 [17.1 (13.7-23.3)] years born from January 1987 who were not exposed to radioactive fallout.

The RAI-R recurrent metastases reoperated at least 6 months after the primary surgery were detected in 60/2,411 (2.5%) patients of whom 46/2,018 (2.3%) were exposed and 14/393 (3.6%) were not exposed to radiation (p=0.155 for the difference in frequencies). On exploratory analysis, no statistical difference in the distribution of clinicopathological characteristics were detected between the radiogenic and sporadic PTC subgroups for the PTs, PMTSs and RMTSs except for the higher frequency of patients of older age at operation in the radiation-exposed subgroup, (**Supplementary Tables 1–3**). Therefore, all cases were pooled into the PT, PMTS and RMTS groups for further analysis. Data collected or generated in the course of this work are shown in **Supplementary Figures 1–3**, and descriptive characteristics of the pooled groups are presented in **Table 1**.

The PTs were characterized by a low frequency of microcarcinomas, 11.7%; 66.7% of tumors had a dominant papillary growth pattern, 45.0% had tall cell, and 6.7% hobnail features, more than 90% of PTs were non-encapsulated. Multifocality was seen in 31.7% of PTs; lymphovascular invasion was frequent, 75%, and so was extrathyroidal extension, 58.3%. Approximately a half of tumors were pT1, 53.3%; lymph node involvement, according to the pathological reports, was registered in 65% of patients (and in 83.0% in whom lymph node dissection was performed). Distant metastasis was detected in 1.7% of patients (1 case). Oncocytic and cystic changes were observed in 81.7% and 25%, respectively. The frequency of BRAF^V600E^ was high, 69.5%, while that of NRAS^Q61R^ was low, 1.7%, (1 case). The median Ki-67 LI was 5.1%, and tumors with Ki-67 LI exceeding 5% accounted for about a half of all cases, 50.8%. Expression of the p16 protein in tumor invasive areas was found in the vast majority of PTs, 98.3%.

#### Resemblance of clinicopathological characteristics between PT, PMTS and RMTS

Availability of the three types of malignant thyroid tissues from the same patient enabled the analysis of clinicopathological similarities between them. To assess the resemblance, we calculated various measures of association, including the odds ratios (ORs, **Table 2**), the agreement coefficients, correlation coefficients, and performed pairwise comparisons (**Supplementary Figure 4**, **Supplementary Tables 4, 5**) and correspondence analysis (**Supplementary Figure 5**) of tumor characteristics. Overall, different statistics aligned well to each other: the lack of association in the conditional logistic regression models were accompanied by the high agreement and correlation coefficients, and statistically insignificant estimate of the difference. Oppositely, statistically significant ORs were likely to be coupled with poor agreement and correlation coefficients, and statistically significant difference.

**Table 2:** Pairwise associations between clinicopathological characteristics of the PTs, PMTSs and RMTSs.

Parameters	OR (95% CI)^a^	p-value^a^	OR (95% CI)	p-value	OR (95% CI)	p-value
	*PTs (ref) and PMTSs*		*PTs (ref) and RMTSs*		*PMTSs (ref) and RMTSs*	
**Dominant growth pattern**	2.299 (1.073-4.926)	**0.032**	1.738 (1.085-2.784)	**0.021**	1.385 (0.540-3.550)	0.498
papillary	0.250 (0.071-0.886)	**0.032**	0.389 (0.162-0.931)	**0.034**	1.000 (0.202-4.955)	1.000
follicular	2.000 (0.366-10.919)	0.423	0.750 (0.168-3.351)	0.706	0.260 (0.000-1.714)	0.125
solid-trabecular	4.500 (0.972-20.827)	0.054	3.000 ((1.191-7.558)	**0.020**	4.000 (0.396-196.990)	0.375
**Histological subtype**	0.747 (0.537-1.040)	0.084	0.785 (0.620-0.993)	**0.043**	1.029 (0.723-1.474)	0.935
papillary	1.667 (0.324-10.732)	0.727	1.200 (0.518-2.777)	0.670	1.250 (0.336-4.655)	0.739
follicular variant	1.500 (0.251-8.977)	0.657	1.000 (0.141-7.099)	1.000	0.414 (0.000-3.472)	0.250
solid-trabecular	13.933 (2.863-inf ^b^)	**9.77E-04**	10.000 (2.429-88.241)	**1.21E-04**	3.000 (0.536-30.393)	0.289
conventional	0.286 (0.094-0.868)	**0.027**	0.364 (0.162-0.817)	**0.014**	0.857 (0.238-2.979)	1.000
rare (tall cell and Warthin-like)	0.400 (0.078-2.062)	0.273	0.250 (0.053-1.177)	0.080	1.000 (0.072-13.796)	1.000
**Tall cell features**	0.500 (0.125-1.999)	0.327	0.900 (0.366-2.215)	0.819	2.000 (0.287-22.110)	0.688
**Hobnail features**	1.000 (0.141-7.099)	1.000	3.000 (0.812-11.081)	0.099	5.000 (0.559-236.488)	0.219
**Extrathyroidal and extranodal extension**	0.059 (0.008-0.442)	**0.006**	0.368 (0.155-0.876)	**0.024**	1.500 (0.356-7.227)	0.754
**Oncocytic changes**	0.260 (0.000-1.714)	0.125	0.857 (0.238-2.979)	1.000	0.750 (0.110-4.433)	1.000
**Cystic changes**	3.333 (0.858-18.849)	0.092	3.332 (1.291-10.143)	**0.009**	3.000 (0.536-30.393)	0.289
**BRAF^V600E^-positive**	NA^c^	NA	NA	NA	NA	NA
**NRAS^Q61R^-positive**	NA	NA	NA	NA	NA	NA
**Ki-67 labeling index**, % group	0.731 (0.331-1.612)	0.437	0.909 (0.496-1.667)	0.758	0.691 (0.293-1.628)	0.398
**p16-positive TEC, invasive areas**	0.414 (0.000-3.472)	0.250	1.000 (0.053-inf)	0.500	2.414 (0.288-inf)	0.250

a exact conditional logistic regression.

b infinity.

c not available (due to the lack of variability).

Numbers in bold indicate statistical significance.

The PTs displayed very weak similarities of their dominant growth patterns and histological subtypes to those of the PMTSs and RMTSs. However, the presence of tall cell and hobnail features in the PTs was rather preserved in the PMTSs and RMTSs. Extrathyroidal tumor extension of the PTs was in a poor correspondence with extranodal extension in the PMTSs and RMTSs. Oncocytic changes were quite consistent between the three tumor tissue types. Cystic changes in the PTs corresponded weakly with the presence of those in the PMTSs and RMTSs. Mutational statuses of the three tumor tissue types were fully concordant. The distributions of Ki-67 LI groups and p16-positivity in the PT TECs corresponded rather weakly to those of the PMTSs and RMTSs.

The clinicopathological characteristics of the PMTSs and RMTSs, in contrast to those of the PTs, were markedly consistent between each other. Except for the Ki-67 LI distribution, whose resemblance in PMTSs and RMTSs was rather weak, all other parameters moderately to nearly perfectly corresponded to each other in the two metastatic tissues.

#### Changes of clinicopathological characteristics from PT to PMTS and RMTS

Comparison of changes from the PT to PMTS and to RMTS (**Table 2**; **Supplementary Figure 4** for pairwise comparisons) demonstrated that the papillary dominant growth pattern was statistically significantly less frequently observed in the PMTSs and RMTSs (OR = 0.250 (0.071-0.886) and 0.389 (0.162-0.931), respectively) than in the PTs. Accordingly, the frequencies of the conventional histological subtype (which has the papillary dominant growth pattern more commonly) was lower in the metastatic (OR = 0.286 (0.094-0.868) and 0.364 (0.162-0.817) for PMTS and RMTSs, respectively) than in the primary tumors. In contrast, the frequencies of the solid-trabecular dominant growth pattern in the PMTSs and RMTSs (OR = 4.500 (0.972-20.827), marginally, and 3.000 (1.191-7.558), respectively) and of the solid-trabecular subtype (OR = 13.933 (2.863-infinity) and 10.000 (2.429-88.241), respectively) were higher than in the PTs. Cystic changes were also more frequent in the metastatic tumors [OR = 3.333 (0.858-18.849), marginally) and 3.332 (1.291-10.143)]. Frequencies of the tall cell and hobnail features which are commonly associated with the aggressive tumor phenotype did not statistically significantly differ between the three tumor tissue types, although the frequency of the latter tended to increase from PMTS to RMTS. Extranodal extension in the metastatic tumors was less frequent than in the PTs (OR = 0.059 (0.008-0.442) for PMTSs and 0.368 (0.155-0.876) for RMTSs). The distributions of Ki-67 LI and p16-positivity did not shift towards the increase in the metastatic tumors as compared to the PTs (all ORs ≤ 1).

Of note, in contrast to comparisons to PTs, there were no statistically significant changes in the clinicopathological parameters between the PMTSs and RMTSs (**Table 2**; **Supplementary Figure 4**), which is consistent with their highly concordant characteristics described above.

#### Risk factors for RAI-R recurrent metastases

Univariate regression analysis revealed a few risk factors for the RAI-R recurrent metastases (**Supplementary Table 6**). Interestingly, a history of radiation exposure [HR = 0.526 (0.283-0.980)] and the higher POC level [HR = 0.987 (0.975-0.999)] reduced the risk, while the longer period of latency of radiogenic PTCs elevated it [HR = 1.117 (1.047-1.192)]. At the same time, radiation dose to the thyroid was not a risk factor [HR = 0.999 (0.997-1.000)]. Among the clinicopathological characteristics, the presence of N1b primary metastases was a risk factor for RAI-R RMTS [HR = 1.777 (1.034-3.053)]; naturally, since N1b could only be pathologically confirmed after the lateral lymph node dissection, this surgical option was a “technical” risk factor [HR = 1.932 (1.141-3.272)]. The greater number of RAI therapy cycles [HR = 1.220 (1.023-1.456)] and, correspondingly, the higher cumulative activity of RAI (HR = 1.000 (1.000-1.000), p = 0.006) were the treatment options associating with elevated risk for RAI-R that are explainable by the continuous attempts to eradicate a recurrent tumor.

The optimized stratified multivariate regression model of risk for RAI-R RMTS included four variables: the POC level, N1b primary metastases, dominant solid-trabecular growth pattern, and cystic changes in the PT (**Table 3**). The model had a moderate predictive performance as judged by the area under the curve [0.736 (0.595-0.797)], the Brier score (0.325), and Harrell’s concordance index (0.658). In agreement with univariate assessment, the N1b primary metastases was the strongest risk factor (HR = 2.430 (1.308-4.666), and POC level, on the contrary, had a protective impact [HR = 0.983 (0.930-0.996)]. Of note, POC level had an independent effect seen as a negligible change in effect size between the univariate and multivariate models (see **Table 3**). When POC was decomposed into its components (thyroid dose, sex, latency period and age at the time of the Chornobyl accident), it appeared that its “protective” effect was not related to radiation exposure *per se* [i.e., the thyroid dose, HR = 0.897 (0.686-1.172)], but to a longer latency period (i.e., time between irradiation and PT development (HR = 1.116 (1.043-1.194), see **Table 3**). POC is inversely associated with the latency period (the longer the latency, the lower POC), again pointing at the validity of POC association with the risk for RMTS due to the period of latency, which was also seen on univariate analysis.

**Table 3:** Risk factors for recurrent RAI-R metastases.

Parameters	HR (95% CI)	p-value
*Optimized multivariate model*		
**Probability of causation (POC)**, %	0.983 (0.970-0.996)	**0.011**
**N1b**	2.470 (1.308-4.666)	**0.005**
**Solid-trabecular growth pattern**	1.899 (0.922-3.910)	0.063
**Cystic changes**	1.483 (0.757-2.906)	0.251
Model performance: AIC^1^ = 372.098; MITD AUC^2^ = 0.736 (0.595-0.797); BS^3^ = 0.325; HC^4^ = 0.658		
*Independent effect of the Probability of causation (POC)*		
**Probability of causation (POC),** %	0.987 (0.975-0.999)	**0.036**
Model performance: AIC=374.200; MITD AUC=0.652 (0.641-0.661); BS=0.328; HC=0.604		
*POC decomposed to the components*		
**Thyroid dose**, mGy (log)	0.897 (0.686-1.172)	0.426
**Sex** (ref=F)	0.788 (0.382-1.625)	0.519
**Period of latency**, years	1.116 (1.043-1.194)	**0.001**
**Age at accident**, years	1.007 (0.937-1.082)	0.857
Model performance: AIC=260.989; MITD AUC=0.779 (0.670-0.868); BS=0.326; HC=0.683		

^1^ Akaike information criterion.

^2^ Median integrated time-dependent area under curve (5-95% distribution-free CI).

^3^ Brier score (a metric of the accuracy of probabilistic prediction; the closer to 0, the better).

^4^ Harrell’s concordance index (predictive power of a model; the closer to 1, the better).

Numbers in bold indicate statistical significance.

Despite the effects of two other variables in the multivariate model, i.e. the solid-trabecular growth pattern and cystic changes did not reach statistical significance, the presence of those in pathological report would be advisable to be taken into account as factors potentially suggestive of the risk for developing RAI-R RMTS.

### Immune checkpoint status

#### Positive ICS *versus* negative ICS

About 50% of TECs and 75-85% of TAICs were positive for PD-L1, and 60-70% of TAICs also expressed PD-1 in the PT, PMTS and RMTS (**Table 4**). Based on the definition (see Methods), positive ICS for each individual tumor tissue was found in 23.7% (14/59) of PTs, 34.3% (12/35) of PMTS, and 28.8% (17/59) of RMTS.

**Table 4:** Immune checkpoint (ICS) status of the primary tumors, primary metastases and recurrent metastases of the RAI-R recurrent PTCs.

Parameters	Primary tumors n=59 (%)	Primary LN metastases n=35 (%)	Recurrent metastases n=59 (%)
**PD-L1-positive TEC** ^1^	30 (50.8%)	19 (54.2%)	29 (49.1%)
≤ 25%	14 (23.7%)	7 (20.0%)	12 (20.3%)
> 25 – 50%	13 (22.0%)	6 (17.1%)	7 (11.9%)
> 50 – 75%	3 (5.1%)	6 (17.1%)	10 (16.9%)
> 75 – 100%	0	0	0
**PD-L1-positive TAIC** ^2^	44 (74.6%)	30 (85.7%)	48 (81.4%)
≤ 25%	35 (59.3%)	17 (48.6%)	28 (47.5%)
> 25 – 50%	9 (15.3%)	13 (37.1%)	20 (33.9%)
> 50 – 75%	0	0	0
> 75 – 100%	0	0	0
**PD-1-positive TAIC**	35 (59.3%)	26 (74.3%)	37 (62.7%)
≤ 25%	31 (52.5%)	16 (45.7%)	29 (49.2%)
> 25 – 50%	4 (6.8%)	10 (28.6%)	8 (13.5%)
> 50 – 75%	0	0	0
> 75 – 100%	0	0	0
**Positive ICS**	14 (23.7%)	12 (34.3%)	17 (28.8%)

^1^ Tumor epithelial cells.

^2^ Tumor-associated immune cells.

Comparisons of baseline and clinicopathological characteristics of ICS-positive and ICs-negative tumor tissues of the PTs, PMTSs and RMTSs are presented in **Supplementary Tables 7–9**, respectively. In general, in all three forms of tumors, most of characteristics did not differ, including a radiation history, yet some were at variance.

**Figure 1:**
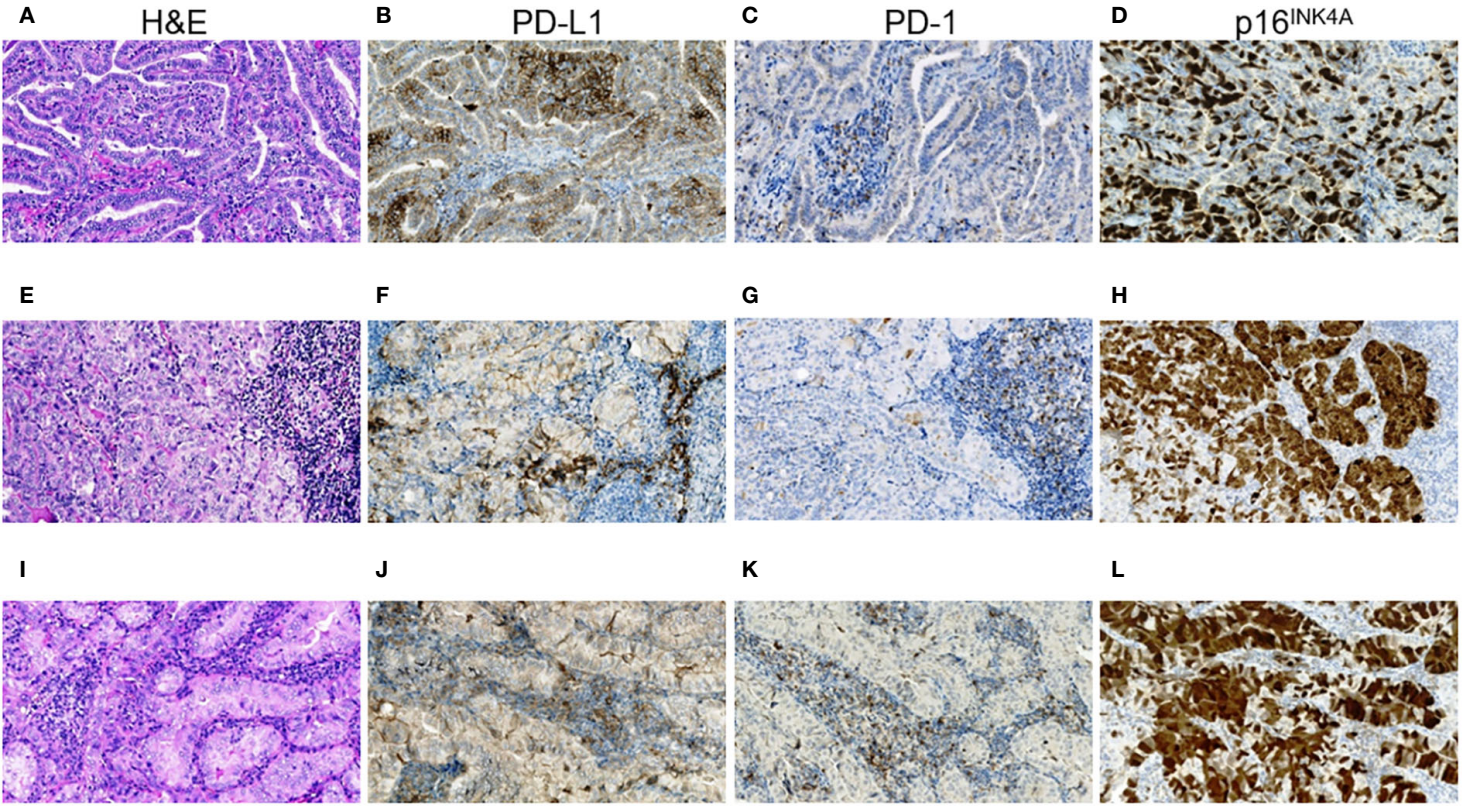
RAI-R recurrent papillary thyroid carcinoma with positive immune checkpoint status and p16^INK4A^ staining. **(A–D)** Primary tumor: **(A)** papillary-trabecular growth pattern, tall cell features, oncocytic changes, H&E, ×200; **(B)** positive membrane-cytoplasmic IHC staining for PD-L1 in > 50% of TECs, some positive TAICs can be seen, ×200; **(C)** negative IHC staining for PD-1 in TECs and positive reaction in up to 25% TAICs, ×200; **(D)** positive nucleo-cytoplasmic IHC staining for p16^INK4A^ in > 50% TECs, ×200. **(E–H)** Primary oncocytic cell metastasis of the tumor: **(E)** solid growth pattern, H&E, ×200; **(F)** positive membrane-cytoplasmic IHC staining for PD-L1 in > 50% of TECs and in > 25% TAICs, ×200; **(G)** negative IHC staining for PD-1 in TECs and positive reaction in > 25% TAICs, ×200; **(H)** positive nucleo-cytoplasmic IHC staining for p16^INK4A^ in > 75% TECs, ×200; **(I–L)** RAI-R recurrent oncocytic cell metastasis removed 2.8 years after the 1^st^ surgery: **(I)** solid-trabecular growth pattern, H&E, ×200; **(J)** positive membrane-cytoplasmic IHC staining for PD-L1 in > 50% of TECs and in > 25% TAICs, ×200; **(K)** negative IHC staining for PD-1 in TECs and positive reaction in > 25% TAICs, ×200; **(L)** positive nucleo-cytoplasmic IHC staining for p16^INK4A^ in > 75% TECs, ×200. H&E, hematoxylin-eosin staining; TECs, tumor epithelial cells; TAICs, tumor-associated immune cells.

In the subgroup of ICS-positive PTs **(Figures 1A-D)**, there were only female patients (p = 0.013), more frequent rare histological variants (p = 0.047, the Warthin-like and tall cell variants), more frequent pronounced oncocytic changes (in > 50% TECs, p = 0.013), higher Ki-67 LI (p = 0.003), and higher frequencies of p16-positivity (in > 50% TECs, p = 0.030) and of chronic thyroiditis (p = 4.41E-04).

In the ICS-positive PMTSs **(Figures 1E-H)**, the differences included more frequent solid-trabecular dominant growth pattern and subtype (p = 0.011 and 0.022, respectively), higher frequency of oncocytic metastatic tumors (p = 0.038) and of p16-positivity (in > 75% TECs, p = 0.007). Ki-67 LI was somewhat higher, but the difference did not reach statistical significance (p = 0.087).

The ICS-positive RMTSs **(Figures 1I-L)** were characterized by the female patient prevalence (p = 0.045), less frequent papillary growth pattern and subtype (p = 3.10E-05 and 0.003, respectively) and, in contrast, more frequent solid-trabecular growth pattern and subtype (p = 2.00E-06 and 2.96E-04, respectively), more frequent pronounced oncocytic changes (in > 50% TECs, p = 1.11E-04), higher Ki-67 LI (p = 0.041), and higher frequency of p16-positivity (in > 50% TECs, p = 0.007).

**Figure 2:**
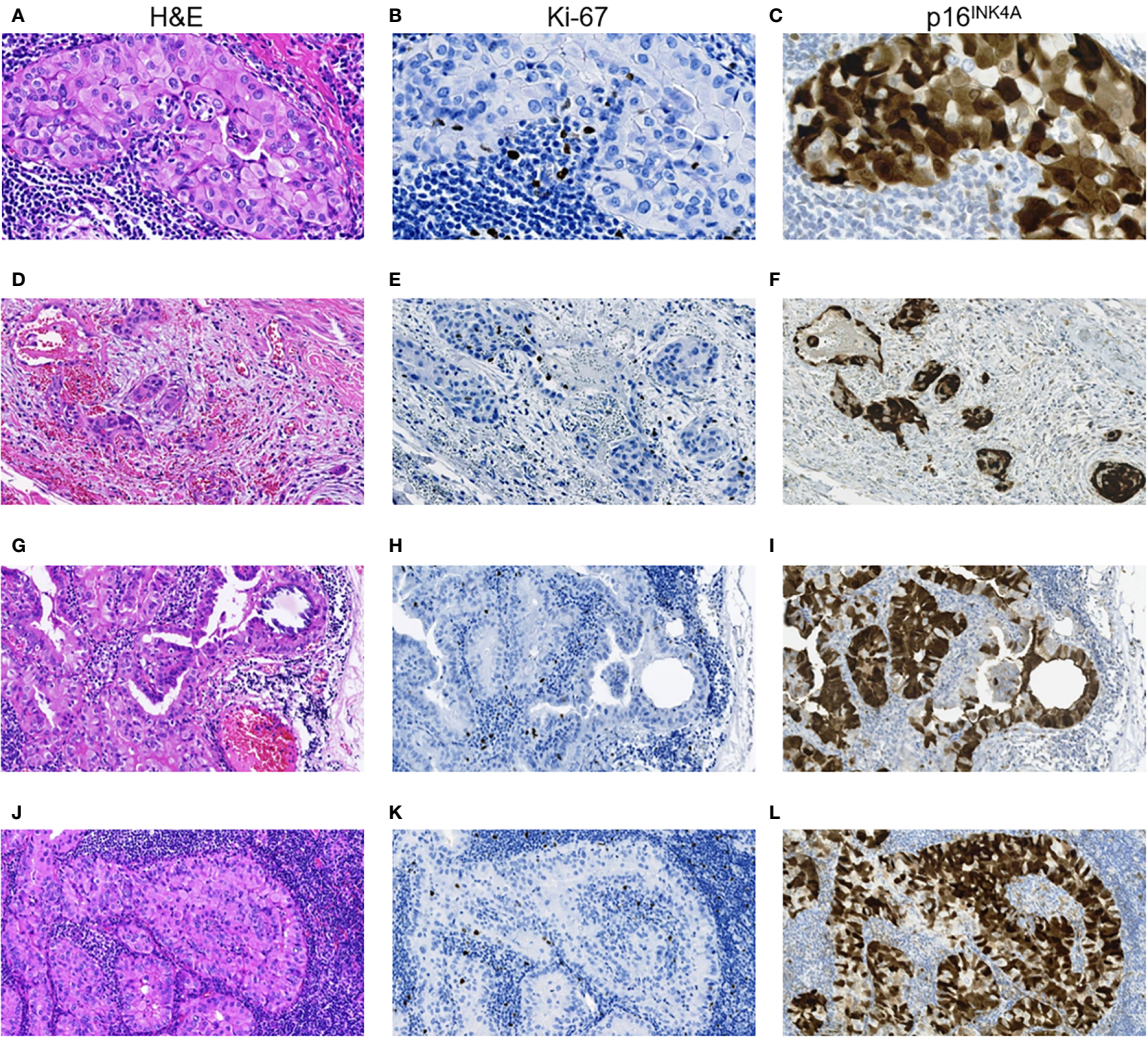
RAI-R recurrent papillary thyroid carcinoma with positive immune checkpoint status: particularities of Ki-67 and p16^INK4A^ expression. **(A–C)** Primary tumor: **(A)** intrathyroid spread, oncocytic solid TEC locus, H&E, ×400; **(B)** same locus, isolated Ki-67 positive TECs, IHC reaction with anti-Ki67 antibody, ×400; **(C)** same locus, positive nucleo-cytoplasmic IHC staining for p16^INK4A^ in > 75% TECs, ×400. **(D–F)** Primary tumor: **(D)** extrathyroidal extension of TEC loci, H&E, ×200; **(E)** same area, isolated Ki-67 positive TECs, IHC reaction with anti-Ki67 antibody, ×200; **(F)** positive nucleo-cytoplasmic IHC staining for p16^INK4A^ in > 75% TECs, ×200. **(G–I)** Primary lymph node metastasis of the tumor: **(G)** peripheral area with solid-papillary structure, H&E, ×400; **(H)** same area, isolated Ki-67 positive TECs, IHC reaction with anti-Ki67 antibody, ×400; **(I)** same area, positive nucleo-cytoplasmic IHC staining for p16^INK4A^ in > 75% TECs, ×400; **(J–L)** RAI-R recurrent lymph node metastasis of the tumor: **(J)** oncocytic solid tumor loci spread, H&E, ×400; **(K)** same area, isolated Ki-67 positive TECs, IHC reaction with anti-Ki67 antibody, ×400; **(L)** same area, positive nucleo-cytoplasmic IHC staining for p16^INK4A^ in > 75% TECs, ×400. H&E, hematoxylin-eosin staining; TEC, tumor epithelial cell.

**Figure 3:**
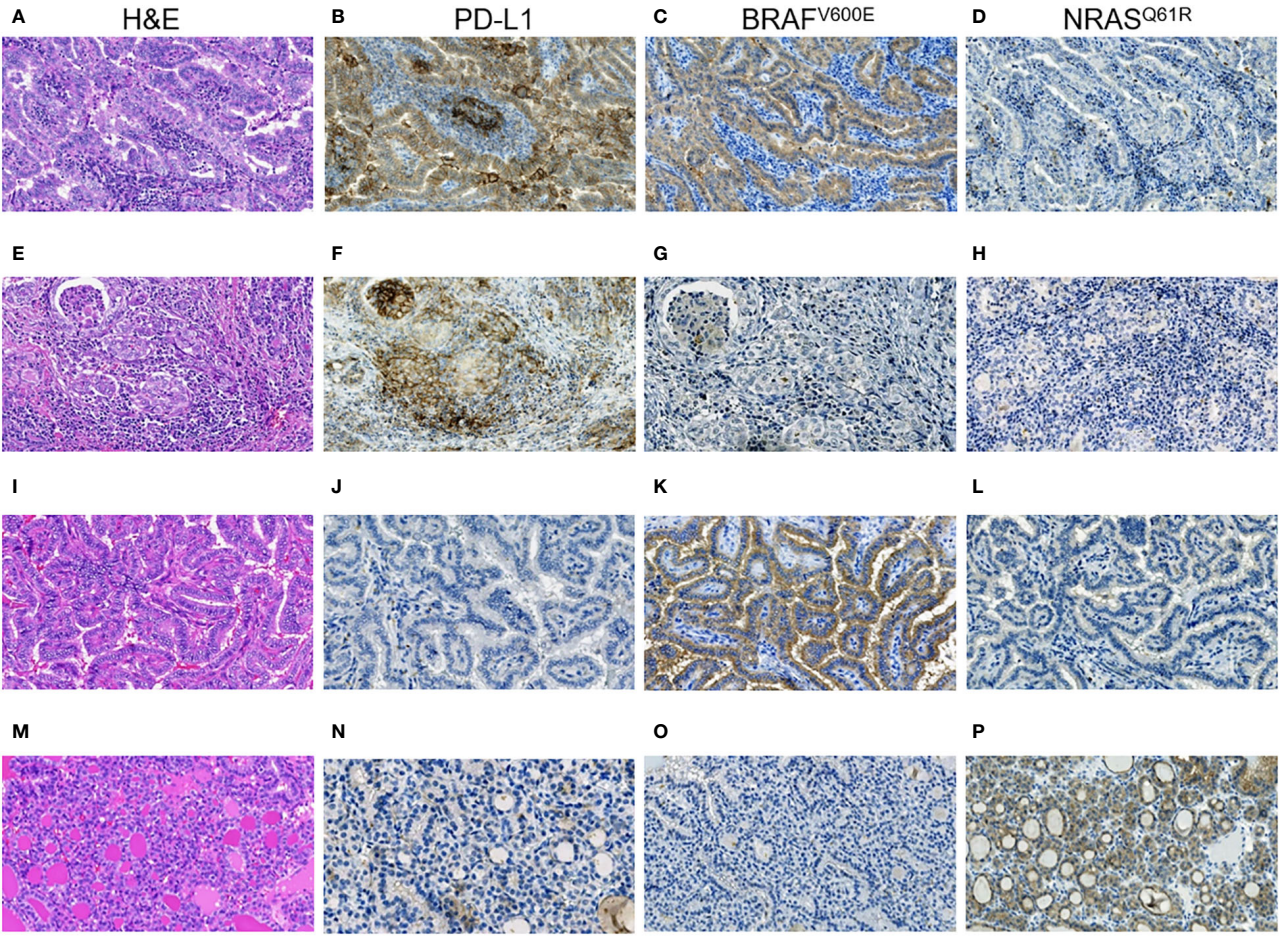
RAI-R recurrent papillary thyroid carcinomas with different immune checkpoint status and driver oncogenes. **(A–D)** Primary tumor: **(A)** Warthin-like growth pattern, oncocytic changes, H&E, ×200; **(B)** positive membrane-cytoplasmic IHC staining for PD-L1 in > 50% of TECs, and in > 25% TAICs, ×200; **(C)** positive diffuse cytoplasmic IHC staining for BRAF^V600E^ in TECs, ×200; **(D)** negative IHC staining for NRAS^Q61R^ in TECs, ×200. **(E-H)** Primary tumor: **(E)** solid growth pattern, oncocytic changes, H&E, ×200; **(F)** positive membrane-cytoplasmic IHC staining for PD-L1 in > 50% of TECs, and in > 25% TAICs, ×200; **(G)** negative IHC staining for BRAF^V600E^ in TECs, ×200; **(H)** negative IHC staining for NRAS^Q61R^ in TECs, ×200. **(I–L)** Primary tumor: **(I)** papillary growth pattern, oncocytic changes, focal tall cell features, H&E, ×200; **(J)** negative IHC staining for PD-L1 in TECs, ×200; **(K)** positive diffuse cytoplasmic IHC staining for BRAF^V600E^ in TECs, ×200; **(L)** negative IHC staining for NRAS^Q61R^ in TECs, ×200. **(M–P)** Primary tumor: **(M)** fully encapsulated, follicular growth pattern, H&E, ×200; **(N)** negative IHC staining for PD-L1 in TECs, ×200; **(O)** negative IHC staining for BRAF^V600E^ in TECs, ×200; **(P)** positive membrane-cytoplasmic staining for NRAS^Q61R^ in TECs, ×200. H&E, hematoxylin-eosin staining; TECs, tumor epithelial cells; TAICs, tumor-associated immune cells.

Note that, while the ICS-positive PTs had comparable proportions of the dominant papillary and solid-trabecular architecture, the PMTSs and especially RMTSs had a solid-trabecular dominant growth pattern with increasing frequencies (**Figures 1A, E, I**). A similar tendency was observed for the p16-positivity in tumor invasive areas (**Figures 1D, H, L**). Also of interest, despite the ICS-positive tumors generally had a higher Ki-67 LI and p16-positivity than the ICS-negative ones, even in the areas with high density of the p16-positive TECs, the Ki67-positive TECs were absent or isolated and did not colocalize with the p16^INK4A^-positive cells (**Figure 2**). No statistically significant correlation between Ki-67 and p16^INK4A^ levels was detected in the primary or metastatic tumors (p > 0.05 for any comparison). No indications pointing at the association between the mutational status and ICS was found in a given group of RAI-R PTCs (**Supplementary Tables 7–9**; **Figure 3**).

#### Predictors of the ICS positivity

**Figure 4:**
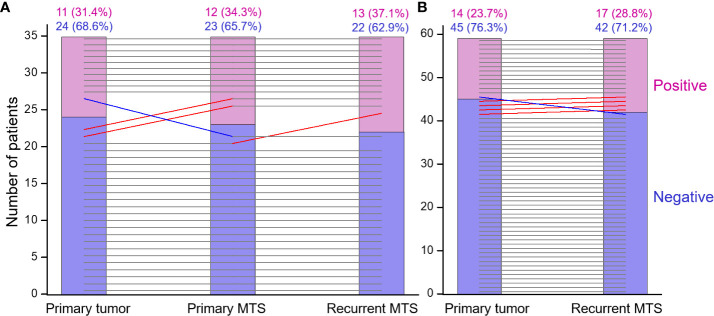
The immune checkpoint statuses of the primary tumor, primary metastasis and recurrent RAI-R metastasis are highly concordant. **(A)** Primary tumor, primary metastasis and recurrent RAI-R metastasis (n=35). **(B)** Primary tumor and recurrent RAI-R metastasis (n=59). The grey horizontal lines indicate no change in the statuses of tumors from the same patient. The red and blue lines indicate status elevation or decline, respectively; the slopes do not reflect the degree of change and serve for depiction only.

First, we assessed the consistency of ICSs of the PTs, PMTSs and RMTSs. As shown in **Table 5**, for the vast majority of cases, there was an excellent agreement, correlation and no statistical difference between ICSs in the three corresponding tumors. There was only one patient in whom the positive ICS of the PT was lost in the PMTS and RMTS (**Figure 4**). In contrast, in four patients with ICS-negative PTs, ICS-positivity was observed in the RMTSs, of whom in two patients ICS-positivity was gained in PMTSs and persisted in RMTSs, and in one patient with ICS-negative PT and PMTS, ICS positivity was found in RMTS. These data strongly suggest that there is no reason to expect frequent decline of ICS in RMTS as compared to that of PT and/or PMTS.

**Table 5:** The immune checkpoint status resemblance in the primary tumors, primary metastases and recurrent metastases.

Parameters	Agreement^a^	Correlation^b^	Comparison^c^
*Primary tumor and primary metastasis*			
Percent agreement	91.40%		
Coefficient (95% CI)	0.829 (0.564-1.000)	0.808 (0.738-0.860)	
p-value	**4.45E-08**	**2.49E-06**	1.000
*Primary tumor and recurrent metastasis*			
Percent agreement	91.5%		
Coefficient (95% CI)	0.831 (0.681-0.980)	0.789 (0.713-0.846)	
p-value	**4.44E-16**	**1.89E-09**	0.688
*Primary metastasis and recurrent metastasis*			
Percent agreement	97.10%		
Coefficient (95% CI)	0.943 (0.706-1.000)	0.940 (0.906-0.962)	
p-value	**6.10E-11**	**4.28E-08**	1.000

a weighted Brennan-Prediger kappa (ordinal).

b Kendall’s tau-b.

c Wilcoxon signed rank test using the Pratt method, exact p-value.

Numbers in bold indicate statistical significance.

A highly consistent ICS between the tumors from the same patient also suggested that ICS of the PT may be a good predictor of ICS of the RMTS. Indeed, a multivariate regression model adjusted for age and sex returned the following parameter estimates for the ICS of the PT: OR = 76.488 (8.201-713.404), p = 1.407E-07, AUC = 0.920 (0.840-1.000), crossvalidated AUC = 0.861 (0.729-0.994), Brier score = 0.075.

Finally, we attempted to determine clinicopathological parameters that could be associated with the positive ICS of the PT, PMTS and RMTS. The optimized multivariate models (**Table 6**) showed that for the PTs these were the pronounced oncocytic changes [OR = 2.235 (1.120-4.460)] and a high density of p16^INK4A^-positive TECs in the invasive tumor areas [OR = 2.582 (1.259-5.292)]. For PMTSs these were the dominant solid-trabecular growth pattern (OR = 48.596 (2.972-794.494)) and Ki-67 LI > 4.3% [OR = 1.676 (1.072-2.621)] of the PMTS. For RMTSs, the pronounced oncocytic changes [OR = 2.921 (1.405-6.074)] and Ki-67 LI > 4.5% [OR = 1.290 (1.033-1.610)] of the PT, and the solid-trabecular growth pattern [OR = 48.596 (2.972-794.494)] and Ki-67 LI > 7.6% [OR = 1.594 (1.095- 2.321)] and a high density of p16^INK4A^-positive TECs in the invasive metastatic areas [OR=3.374 (1.169-9.739)] of the RMTS were the predictors. Note that the formal risk scores (i.e., the odds ratios of ICS positivity of a given tumor tissue depending on the number of the identified by the model predictors) were consistently increasing with increasing number of such predictors (see **Table 6**). These findings may be useful for tentative or preliminary determination of candidate patients with RAI-R PTC for ICS testing if deemed necessary.

**Table 6:** Factors associated with the positive immune checkpoint status of the primary tumors, primary metastases and recurrent metastases.

Parameters	OR (95% CI)	p-value	Cut-off^1^
*Primary tumors (primary tumor parameters)*			
**Oncocytic changes**	2.235 (1.120-4.460)	**0.022**	Severe/oncocytic tumor
**p16 positivity in invasive areas**	2.582 (1.259-5.292)	**0.010**	≥ 50% p16-positive cells
Model performance: AIC^2^ = 56.583; AUC^2^ = 0.797 (0.647-0.947); AUC-CV^4^ = 0.716 (0.538-0.894); BS^5^ = 0.132			
Parameters present^6^			
**0**	1.000	ref	
**1**	4.000 (0.721-22.183)	0.113	
**2**	24.000 (3.247-177.404)	**0.002**	
*Primary metastases (primary metastasis parameters)*			
**Solid-trabecular growth pattern**	48.596 (2.972-794.494)	**0.006**	Sol-Trab structures ≥ 50%
**Ki-67 labeling index**	1.676 (1.072-2.621)	**0.024**	Ki-67 LI ≥ 4.3%
Model performance: AIC=33.183; AUC=0.881 (0.769-0.993); AUC-CV=0.818 (0.675-0.962); BS=0.134			
Parameters present			
**0**	1.000	ref	
**1**	9.881 (0.423-231.015)	0.154	
**2**	82.347 (2.370-inf)	**0.015**	
*Recurrent RAI-R metastases (primary tumor parameters)*			
**Oncocytic changes**	2.921 (1.405-6.074)	**0.004**	Severe/oncocytic tumor
**Ki-67 labeling index**	1.290 (1.033-1.610)	**0.024**	KI-67 LI ≥ 4.5%
Model performance: AIC=60.355; AUC=0.807 (0.680-0.934); AUC-CV=0.754 (0.613-0.894); BS=0.150			
Parameters present			
**0**	1.000	ref	
**1**	6.856 (0.781-60.159)	0.082	
**2**	47.990 (4.304-535.055)	**0.002**	
*Recurrent RAI-R metastases (recurrent RAI-R metastasis parameters)*			
**Solid-trabecular growth pattern**	43.635 (5.075-375.185)	**0.001**	Sol-Trab structures ≥ 50%
**Ki-67 labeling index**	1.594 (1.095-2.321)	**0.014**	KI-67 LI ≥ 7.6%
**p16 positivity in invasive areas**	3.374 (1.169-9.739)	**0.025**	≥ 75% p16-positive cells
Model performance: AIC=28.527; AUC=0.969 (0.931-1.000); AUC-CV=0.931 (0.855-1.000); BS=0.067			
Parameters present			
**0**	1.000	ref	
**1**	6.067 (0.273-134.747)	0.254	
**2**	246.999 (8.247-inf)	**0.001**	
**3**	428.949 (5.915-inf)	**0.006**	

^1^ Cut-off values were determined using ROC analysis.

^2^ Akaike information criterion.

^3^ Area under the curve.

^4^ Leave-one-out cross-validated area under the curve.

^5^ Brier score.

^6^ Tumors were categorized by the unweighted number of parameters from the corresponding regression model.

Numbers in bold indicate statistical significance.

## Discussion

The first major purpose of this study was to determine the prevalence of the RAI-R recurrent metastases in young and middle-aged PTC patients, and whether radiation exposure in childhood and/or other clinicopathological characteristics affect the chance of such recurrences.

We found that in young and middle-aged patients the RAI-R RMTSs of PTC are rather rare (~2-4%). This frequency is somewhat lower than that reported in the literature (8–10), and it is explainable by a relatively young age of patients in the CTB. The frequency of RAI-R RMTSs was low among both exposed to radiation in childhood and non-exposed individuals. The difference between the frequency of RAI-R RMTSs in the two etiological groups was negligible despite the exposed patients being significantly older at first surgery. The unlikeliness that radiation exposure may specifically affect the risk of developing RAI-R RMTS was further confirmed by regression models which demonstrated statistically insignificant impact of thyroid radiation dose.

Our analysis of histopathological resemblance of PT and metastatic tissues from the same patient clearly showed that PTs were quite different from metastases, whereas PMTSs and RMTSs displayed a high degree of similarity. Structural differences between primary PTC and their PMTS are well described, and usually a classical papillary structure is more often observed in PMTS than in PT (43), that is, metastatic lesions more often have a more differentiated phenotype than the PT. In our study, in contrast, both PMTS and RAI-R RMTS differed from PT by a more frequent less differentiated phenotype, namely a solid-trabecular structure, suggestive of a higher metastatic potential of TECs from such areas of PTs than that of cells from tumor areas of other structure. The current study was focused on RAI-R cases, however, which are the minority of PTCs that may demonstrate difference from PTCs totality.

In line with the latter observation, the dominant solid-trabecular growth pattern of PT was a parameter remaining (although with the borderline statistical significance) in the optimized risk model (see **Table 3**), and therefore it can be considered as a warning histopathological characteristic suggestive of the possibility of further development of RAI-R RMTS. While radiation dose to the thyroid did not appear to be a risk factor for recurrence, the longer latency period did associate with the RAI-R RMTSs risk, demonstrating that the chance of RMTS development is proportional to the time factor. A statistically significant parameter in our study that increased the risk of RAI-R was the lateral primary metastasis (N1b), which was also reported among risk factors for older patients (18).

The *BRAF^V600E^* mutation has been claimed to be a risk factor for recurrent RAI-R metastases of PTC in a number of studies (12, 16, 44, 45). In our previous work, we also obtained similar results in subjects exposed to radiation in childhood who lived in the northern regions of Ukraine most affected by the Chornobyl accident (38). The presence of the *BRAF^V600E^* mutation was associated with the decreased POC level and longer latency. We noted that among the 8 PTC cases with RAI-R RMTSs in the mentioned paper, 6 (75%) were BRAF^V600E^-positive. In the current study, we analyzed the RAI-R RMTSs among all CTB donors with radiogenic and sporadic PTC and obtained almost the same frequency of BRAF^V600E^-positive cases (41/59, 69.5%, p = 1.000 compared to the previous work). From analytical point of view, the current study included RAI-R RMTS-only cases, which makes statistical models in it principally different from previous works which included both RAI-R RMTS and non-RAI-R RMTS cases. The difference in data sets and study design are the likeliest reasons for the presence or absence of certain other parameters in the presented multivariate models, which need to be interpreted appropriately. Despite the fact that *BRAF^V600E^* mutation was not in the optimized risk model in the current study, it still should be considered as a factor affecting the chance of RAI-R RMTS. In practice, oncocytic changes in TECs are strongly predictive of the BRAF^V600E^ mutation, and therefore can be used as a morphological indicator suggestive of the presence of this oncogenic driver both in radiation-related and sporadic PTC (34, 38).

Our second purpose was to determine how often the positive immune checkpoint status can be expected in the primary tumors and metastatic tissues, and which clinicopathological characteristics are associated with it.

ICS positivity was found in about one-quarter of primary or metastatic tumors. This is not an exceedingly small proportion, and may thus deserve attention from clinicians. Note that ICS percent agreement between the PTs and metastatic tissues was 91%, and that between metastatic tissues was as high as 97%. This finding strongly suggests that ICS is rather consistent between a given PT and its metastases in most cases, and that ICS of a PT may be a strong predictor of ICS of a RMTS. In the current tumor series only one ICS-positive PT gave rise to ICS-negative PMTS and RMTS. On the contrary, ICS elevation from PT to RMTS was noted in 4 cases, demonstrating that the loss of ICS positivity in metastatic tissues is highly unlikely.

Positive ICS of primary and metastatic tumors was not associated with radiation exposure or radiation dose to the thyroid. Statistically significant factors associating with positive ICS of the PTs were more frequent pronounced oncocytic changes and a high density of p16^INK4A^ positive TECs in invasive tumor areas. The p16^INK4A^-positive TECs constitute the so-called senescent tumor cells, which have received considerable attention in PTC during recent years (46–50). Due to their particular senescence-associated secretory phenotype, senescent tumor cells may potentiate tumor invasiveness, cell migration and metastatic spread contributing to PTC progression. Our study found that the high density of p16^INK4A^-positive TECs in invasive areas was associated with the positive ICS both in the primary tumor and in RAI-R recurrent metastases. The mechanism by which tumor senescent cells modulate ICS in PTC is unknown, and remains to be established. Nevertheless, the high p16^INK4A^-positivity of TECs along with pronounced oncocytic changes may serve as useful characteristics of a positive ICS of PT. In addition to pronounced oncocytic changes, Ki-67 LI ≥ 4.5% of PT can also be considered a factor associating with ICS positivity of the RMTS.

It should be noted that in contrast to other studies (51, 52), we did not find an association between positive ICS and the *BRAF^V600E^* mutation. A high frequency of BRAF*^V600E^*-positive RAI-R recurrent PTCs (approximately 70%) was found regardless of their ICS. This difference can probably be due to specific characteristics of a focused group of PTCs in our study restricted to RAI-R recurrent cases in patents of relatively young age. In fact, our study does not disprove the possibility of a link between *BRAF^V600E^* and PD-L1 expression in PTC described by other groups.

In summary, we report that in young and middle-aged patients, the RAI-R recurrent PTCs are rather rare (2.5% of cases). Radiation dose to the thyroid does not appear to elevate the chance for recurrence after the first surgery, although in patients exposed to radiation the longer period of latency does. The presence of lateral primary metastases (N1b) is a risk factor for recurrence, and the solid-trabecular growth pattern and cystic changes in PTs are suggestive risk factors. Histopathological resemblance between PTs and metastatic tumors is rather weak, except for the solid-trabecular structures which tend to be preserved from PTs to metastatic tissues, implying their possibly higher metastatic potential. In contrast, histopathological characteristics of PMTSs and RMTSs are largely concordant, suggesting that only some PT cells may be associated with the development of RAI-R recurrent metastases, at least in the age group studied. About one-fourth of the PTs, PMTSs and RMTSs from young and middle-aged patients with RAI-R recurrent PTCs have positive ICS, which is highly concordant between the primary and metastatic tumors in most cases. ICS positivity is associated with pronounced oncocytic changes and high density of p16^INK4A^ positive TECs in invasive areas of the PTs and RMTSs. Pronounced oncocytic changes in TECs and a high Ki-67 LI of the PTs, and the solid-trabecular growth pattern and a high Ki-67 LI of the RMTSs are also associated with the positive ICS of RMTSs. These data may be useful for the development of individual treatment approaches to a subset of patients with RAI-R recurrent PTC for whom immunotherapy may be considered as an option from which the patients may benefit.

## Data availability statement

The original contributions presented in the study are included in the article/**Supplementary Material**. Further inquiries can be directed to the corresponding author.

## Ethics statement

The studies involving humans were approved by IEM Bioethics Committee and Ethics Committee of Nagasaki University. The studies were conducted in accordance with the local legislation and institutional requirements. The human samples used in this study were acquired from the pathological archive of IEM. Written informed consent for participation was not required from the participants or the participants’ legal guardians/next of kin in accordance with the national legislation and institutional requirements.

## Author contributions

TB: Conceptualization, Data curation, Formal analysis, Funding acquisition, Investigation, Methodology, Visualization, Writing – original draft, Writing – review & editing. TIR: Conceptualization, Data curation, Formal analysis, Funding acquisition, Investigation, Methodology, Resources, Visualization, Writing – original draft, Writing – review & editing. LZ: Conceptualization, Data curation, Formal analysis, Funding acquisition, Investigation, Methodology, Visualization, Writing – review & editing. NM: Conceptualization, Funding acquisition, Methodology, Project administration, Resources, Supervision, Writing – review & editing. MT: Conceptualization, Methodology, Project administration, Supervision, Writing – review & editing. MI: Data curation, Formal analysis, Investigation, Methodology, Writing – review & editing. MB: Data curation, Formal analysis, Methodology, Writing – review & editing. SC: Data curation, Methodology, Writing – review & editing. SG: Data curation, Methodology, Writing – review & editing. SM: Data curation, Writing – review & editing. SY: Conceptualization, Methodology, Writing – review & editing. VAS: Conceptualization, Data curation, Formal analysis, Funding acquisition, Investigation, Methodology, Project administration, Resources, Supervision, Visualization, Writing – original draft, Writing – review & editing.

## References

[1] Aschebrook-Kilfoy B Ward MH Sabra MM Devesa SS. Thyroid cancer incidence patterns in the United States by histologic type, 1992-2006. Thyroid (2011) 21(2):125–34. doi: 10.1089/thy.2010.0021 PMC3025182 21186939

[2] Dal Maso L Tavilla A Pacini F Serraino D van Dijk BAC Chirlaque MD. Survival of 86,690 patients with thyroid cancer: A population-based study in 29 European countries from Eurocare-5. Eur J Cancer (2017) 77:140–52. doi: 10.1016/j.ejca.2017.02.023 28410490

[3] Baloch ZW Asa SL Barletta JA Ghossein RA Juhlin CC Jung CK. Overview of the 2022 who classification of thyroid neoplasms. Endocr Pathol (2022) 33(1):27–63. doi: 10.1007/s12022-022-09707-3 35288841

[4] Haugen BR Alexander EK Bible KC Doherty GM Mandel SJ Nikiforov YE. 2015 American thyroid association management guidelines for adult patients with thyroid nodules and differentiated thyroid cancer: the american thyroid association guidelines task force on thyroid nodules and differentiated thyroid cancer. Thyroid (2016) 26(1):1–133. doi: 10.1089/thy.2015.0020 26462967 PMC4739132

[5] Luster M Aktolun C Amendoeira I Barczynski M Bible KC Duntas LH. European perspective on 2015 american thyroid association management guidelines for adult patients with thyroid nodules and differentiated thyroid cancer: proceedings of an interactive international symposium. Thyroid (2019) 29(1):7–26. doi: 10.1089/thy.2017.0129 30484394

[6] Tuttle RM Ahuja S Avram AM Bernet VJ Bourguet P Daniels GH. Controversies, consensus, and collaboration in the use of (131)I therapy in differentiated thyroid cancer: A joint statement from the American Thyroid Association, the European Association of Nuclear Medicine, the Society of Nuclear Medicine and Molecular Imaging, and the European Thyroid Association. Thyroid (2019) 29(4):461–70. doi: 10.1089/thy.2018.0597 30900516

[7] Gulec SA Ahuja S Avram AM Bernet VJ Bourguet P Draganescu C. A joint statement from the American thyroid association, the European association of nuclear medicine, the European thyroid association, the society of nuclear medicine and molecular imaging on current diagnostic and theranostic approaches in the management of thyroid cancer. Thyroid (2021) 31(7):1009–19. doi: 10.1089/thy.2020.0826 33789450

[8] Xing M Haugen BR Schlumberger M. Progress in molecular-based management of differentiated thyroid cancer. Lancet (2013) 381(9871):1058–69. doi: 10.1016/S0140-6736(13)60109-9 PMC3931461 23668556

[9] Worden F. Treatment strategies for radioactive iodine-refractory differentiated thyroid cancer. Ther Adv Med Oncol (2014) 6(6):267–79. doi: 10.1177/1758834014548188 PMC4206652 25364392

[10] Capdevila J Galofre JC Grande E Zafon Llopis C Ramon YCAT Navarro Gonzalez E. Consensus on the management of advanced radioactive iodine-refractory differentiated thyroid cancer on behalf of the Spanish society of endocrinology thyroid cancer working group (Gtseen) and Spanish rare cancer working group (Gethi). Clin Transl Oncol (2017) 19(3):279–87. doi: 10.1007/s12094-016-1554-5 27704399

[11] Nixon IJ Whitcher MM Palmer FL Tuttle RM Shaha AR Shah JP. The impact of distant metastases at presentation on prognosis in patients with differentiated carcinoma of the thyroid gland. Thyroid (2012) 22(9):884–9. doi: 10.1089/thy.2011.0535 PMC3714454 22827579

[12] Aashiq M Silverman DA Na’ara S Takahashi H Amit M. Radioiodine-refractory thyroid cancer: molecular basis of redifferentiation therapies, management, and novel therapies. Cancers (Basel) (2019) 11(9):1382. doi: 10.3390/cancers11091382 31533238 PMC6770909

[13] Durante C Haddy N Baudin E Leboulleux S Hartl D Travagli JP. Long-term outcome of 444 patients with distant metastases from papillary and follicular thyroid carcinoma: benefits and limits of radioiodine therapy. J Clin Endocrinol Metab (2006) 91(8):2892–9. doi: 10.1210/jc.2005-2838 16684830

[14] Schlumberger M Brose M Elisei R Leboulleux S Luster M Pitoia F. Definition and management of radioactive iodine-refractory differentiated thyroid cancer. Lancet Diabetes Endocrinol (2014) 2(5):356–8. doi: 10.1016/S2213-8587(13)70215-8 24795243

[15] Schmidt A Iglesias L Klain M Pitoia F Schlumberger MJ. Radioactive iodine-refractory differentiated thyroid cancer: an uncommon but challenging situation. Arch Endocrinol Metab (2017) 61(1):81–9. doi: 10.1590/2359-3997000000245 PMC10522117 28225999

[16] Liu H Yang D Li L Tu Y Chen C Sun S. Appraisal of radioiodine refractory thyroid cancer: advances and challenges. Am J Cancer Res (2020) 10(7):1923–36. PMC7407348 32774993

[17] Nakanishi K Kikumori T Miyajima N Takano Y Noda S Takeuchi D. Impact of patient age and histological type on radioactive iodine avidity of recurrent lesions of differentiated thyroid carcinoma. Clin Nucl Med (2018) 43(7):482–5. doi: 10.1097/RLU.0000000000002078 29688947

[18] Shi LY Liu J Yu LJ Lei YM Leng SX Zhang HY. Clinic-pathologic features and prognostic analysis of thyroid cancer in the older adult: A seer based study. J Cancer (2018) 9(15):2744–50. doi: 10.7150/jca.24625 PMC6072817 30087716

[19] Saie C Wassermann J Mathy E Chereau N Leenhardt L Tezenas du Montcel S. Impact of age on survival in radioiodine refractory differentiated thyroid cancer patients. Eur J Endocrinol (2021) 184(5):667–76. doi: 10.1530/EJE-20-1073 33667193

[20] Thomas GA Williams ED Becker DV Bogdanova TI Demidchik EP Lushnikov E. Chernobyl tumor bank. Thyroid (2000) 10(12):1126–7. doi: 10.1089/thy.2000.10.1126a 11201862

[21] Thomas GA. The chernobyl tissue bank: integrating research on radiation-induced thyroid cancer. J Radiol Prot (2012) 32(1):N77–80. doi: 10.1088/0952-4746/32/1/N77 22394998

[22] Nervo A Retta F Ragni A Piovesan A Gallo M Arvat E. Management of progressive radioiodine-refractory thyroid carcinoma: current perspective. Cancer Manag Res (2022) 14:3047–62. doi: 10.2147/CMAR.S340967 PMC9584766 36275786

[23] Shi RL Qu N Luo TX Xiang J Liao T Sun GH. Programmed death-ligand 1 expression in papillary thyroid cancer and its correlation with clinicopathologic factors and recurrence. Thyroid (2017) 27(4):537–45. doi: 10.1089/thy.2016.0228 27825291

[24] Ulisse S Tuccilli C Sorrenti S Antonelli A Fallahi P D’Armiento E. Pd-1 ligand expression in epithelial thyroid cancers: potential clinical implications. Int J Mol Sci (2019) 20(6):1405. doi: 10.3390/ijms20061405 30897754 PMC6471477

[25] D’Andrea G Lassalle S Guevara N Mograbi B Hofman P. From biomarkers to therapeutic targets: the promise of pd-L1 in thyroid autoimmunity and cancer. Theranostics (2021) 11(3):1310–25. doi: 10.7150/thno.50333 PMC7738901 33391536

[26] Kovacevic B Vucevic D Cerovic S Eloy C. Peripheral versus intraparenchymal papillary thyroid microcarcinoma: different morphologies and pd-L1 expression. Head Neck Pathol (2022) 16(1):200–12. doi: 10.1007/s12105-021-01337-1 PMC9018942 34076845

[27] Oh DY Algazi A Capdevila J Longo F Miller W Jr. Chun Bing JT. Efficacy and safety of pembrolizumab monotherapy in patients with advanced thyroid cancer in the phase 2 keynote-158 study. Cancer (2023) 129(8):1195–204. doi: 10.1002/cncr.34657 36748723

[28] Varricchi G Loffredo S Marone G Modestino L Fallahi P Ferrari SM. The immune landscape of thyroid cancer in the context of immune checkpoint inhibition. Int J Mol Sci (2019) 20(16):3934. doi: 10.3390/ijms20163934 31412566 PMC6720642

[29] Girolami I Pantanowitz L Mete O Brunelli M Marletta S Colato C. Programmed death-ligand 1 (Pd-L1) is a potential biomarker of disease-free survival in papillary thyroid carcinoma: A systematic review and meta-analysis of pd-L1 immunoexpression in follicular epithelial derived thyroid carcinoma. Endocr Pathol (2020) 31(3):291–300. doi: 10.1007/s12022-020-09630-5 32468210

[30] Fullmer T Cabanillas ME Zafereo M. Novel therapeutics in radioactive iodine-resistant thyroid cancer. Front Endocrinol (Lausanne) (2021) 12:720723. doi: 10.3389/fendo.2021.720723 34335481 PMC8321684

[31] Lloyd RV Osamura RY Kloppel G Rosai J. Who Classification of Tumours of Endocrine Organs. 4 ed. Lyon: IARC Press (2017).

[32] Williams ED. Guest editorial: two proposals regarding the terminology of thyroid tumors. Int J Surg Pathol (2000) 8(3):181–3. doi: 10.1177/106689690000800304 11493987

[33] Brierley JD Gospodarowich MK Wittekind C. TNM Classification of Malignant Tumours. 8 ed. Oxford: Wiley-Blackwell (2017).

[34] Zurnadzhy L Bogdanova T Rogounovitch TI Ito M Tronko M Yamashita S. The Braf(V600e) mutation is not a risk factor for more aggressive tumor behavior in radiogenic and sporadic papillary thyroid carcinoma at a young age. Cancers (Basel) (2021) 13(23):6038. doi: 10.3390/cancers13236038 34885148 PMC8656579

[35] Nakao T Matsuse M Saenko V Rogounovitch T Tanaka A Suzuki K. Preoperative detection of the tert promoter mutations in papillary thyroid carcinomas. Clin Endocrinol (Oxf) (2021) 95(5):790–9. doi: 10.1111/cen.14567 34322882

[36] Likhtarov I Thomas G Kovgan L Masiuk S Chepurny M Ivanova O. Reconstruction of individual thyroid doses to the Ukrainian subjects enrolled in the chernobyl tissue bank. Radiat Prot Dosimetry (2013) 156(4):407–23. doi: 10.1093/rpd/nct096 23595409

[37] Kocher DC Apostoaei AI Henshaw RW Hoffman FO Schubauer-Berigan MK Stancescu DO. Interactive radioepidemiological program (Irep): A web-based tool for estimating probability of causation/assigned share of radiogenic cancers. Health Phys (2008) 95(1):119–47. doi: 10.1097/01.HP.0000291191.49583.f7 PMC4018571 18545036

[38] Zurnadzhy L Bogdanova T Rogounovitch TI Ito M Tronko M Yamashita S. Clinicopathological implications of the braf (V600e) mutation in papillary thyroid carcinoma of ukrainian patients exposed to the chernobyl radiation in childhood: A study for 30 years after the accident. Front Med (Lausanne) (2022) 9:882727. doi: 10.3389/fmed.2022.882727 35665338 PMC9159157

[39] Bogdanova T Chernyshov S Zurnadzhy L Rogounovitch TI Mitsutake N Tronko M. The relationship of the clinicopathological characteristics and treatment results of post-chornobyl papillary thyroid microcarcinomas with the latency period and radiation exposure. Front Endocrinol (Lausanne) (2022) 13:1078258. doi: 10.3389/fendo.2022.1078258 36589808 PMC9796818

[40] Likhtarev IA Sobolev BG Kairo IA Tronko ND Bogdanova TI Oleinic VA. Thyroid cancer in the Ukraine. Nature (1995) 375(6530):365. doi: 10.1038/375365a0 7760928

[41] Saenko V Ivanov V Tsyb A Bogdanova T Tronko M Demidchik Y. The chernobyl accident and its consequences. Clin Oncol (R Coll Radiol) (2011) 23(4):234–43. doi: 10.1016/j.clon.2011.01.502 21345660

[42] Tronko M Bogdanova T Saenko V Thomas GA Likhtaterv Yamashita S. Thyroid Cancer in Ukraine after Chernobyl: Dosimetry, Epidemiology, Pathology, Molecular Biology. Nagasaki: IN-TEX (2014).

[43] Nikiforov YE Biddinger PW Thompson LDR. Diagnostic Pathology and Molecular Genetics of the Thyroid: A Comprehensive Guide for Practicing Thyroid Pathology. 3 ed. Philadelphia: Wolters Kluwer (2020).

[44] Luo Y Jiang H Xu W Wang X Ma B Liao T. Clinical, pathological, and molecular characteristics correlating to the occurrence of radioiodine refractory differentiated thyroid carcinoma: A systematic review and meta-analysis. Front Oncol (2020) 10:549882. doi: 10.3389/fonc.2020.549882 33117686 PMC7561400

[45] Jafri S Yaqub A. Redifferentiation of braf V600e-mutated radioiodine refractory metastatic papillary thyroid cancer after treatment with Dabrafenib and Trametinib. Cureus (2021) 13(8):e17488. doi: 10.7759/cureus.17488 34595070 PMC8465644

[46] Zafon C Obiols G Castellvi J Ramon y Cajal S Baena JA Mesa J. Expression of P21cip1, P27kip1, and P16ink4a cyclin-dependent kinase inhibitors in papillary thyroid carcinoma: correlation with clinicopathological factors. Endocr Pathol (2008) 19(3):184–9. doi: 10.1007/s12022-008-9037-z 18766473

[47] Wang P Pei R Lu Z Rao X Liu B. Methylation of P16 cpg islands correlated with metastasis and aggressiveness in papillary thyroid carcinoma. J Chin Med Assoc (2013) 76(3):135–9. doi: 10.1016/j.jcma.2012.11.007 23497965

[48] Kim YH Choi YW Lee J Soh EY Kim JH Park TJ. Senescent tumor cells lead the collective invasion in thyroid cancer. Nat Commun (2017) 8:15208. doi: 10.1038/ncomms15208 28489070 PMC5436223

[49] Yoo SK Song YS Lee EK Hwang J Kim HH Jung G. Integrative analysis of genomic and transcriptomic characteristics associated with progression of aggressive thyroid cancer. Nat Commun (2019) 10(1):2764. doi: 10.1038/s41467-019-10680-5 31235699 PMC6591357

[50] Hong K Cen K Chen Q Dai Y Mai Y Guo Y. Identification and validation of a novel senescence-related biomarker for thyroid cancer to predict the prognosis and immunotherapy. Front Immunol (2023) 14:1128390. doi: 10.3389/fimmu.2023.1128390 36761753 PMC9902917

[51] Siraj AK Parvathareddy SK Pratheeshkumar P Divya SP Al-Sobhi SS Al-Dayel F. Pd-L1 is an independent prognostic marker in middle eastern Ptc and its expression is upregulated by brafv600e mutation. Cancers (Basel) (2021) 13(3):555. doi: 10.3390/cancers13030555 33535609 PMC7867170

[52] Zhang M Gu J Wang W Wang K Zheng L Feng J. Combined expression of the braf(V600e) mutation and Pd-L1 in early papillary thyroid carcinoma and its relationship with clinicopathological features and recurrence-a retrospective cohort study. Gland Surg (2022) 11(12):1908–23. doi: 10.21037/gs-22-701 PMC9840988 36654945

