# Supplementary material for: Characteristics and immune checkpoint status of radioiodine-refractory recurrent papillary thyroid carcinomas from Ukrainian Chornobyl Tissue Bank donors
Source: Front Endocrinol (Lausanne). 2024 Jan 8;14:1343848. doi: 10.3389/fendo.2023.1343848 (PMC10800488; doi:10.3389/fendo.2023.1343848)
Supplement: Supplementary file 1 [file Table_1.docx]

**Supplementary Table 1** Comparison of the radiogenic and sporadic primary tumors

| **Parameter** | **Sporadic, n=14** | **Radiogenic, n=46** | **OR (95% CI)** | **p-value** |
| --- | --- | --- | --- | --- |
| **Sex F/M** (%M); (ref=F) | 11/3 (21.4%) | 33/13 (28.3%) | 1.444 (0.346-6.029) | 0.614 |
| **Age at operation**, years | 17.1 (8.7-25.3; 13.1-23.4) | 32.7 (18.0-48.4; 25.1-36.5) | 1.388 (1.147-1.678) | **0.001** |
| **Tumor size**, mm | 19 (6-55; 14-30) | 20 (6-105; 14-35) | 1.012 (0.973-1.052) | 0.545 |
| **Dominant growth pattern** |  |  | 1.164 (0.566-2.391) | 0.680 |
| **Histological subtype** |  |  | 1.358 (0.951-1.939) | 0.093 |
| **Tall cell features** | 3 (21.4%) | 24 (52.2%) | 4.000 (0.985-16.246) | 0.053 |
| **Hobnail features** | 2 (14.3%) | 2 (4.3%) | 0.273 (0.035-2.143) | 0.217 |
| **Full tumor capsule** | 1 (7.1%) | 3 (6.5%) | 0.907 (0.087-9.478) | 0.935 |
| **Multifocality** | 2 (14.3%) | 17 (37.0%) | 3.517 (0.701-17.635) | 0.126 |
| **Lymphatic/vascular invasion** | 10 (71.4%) | 35 (76.1%) | 1.273 (0.332-4.875) | 0.725 |
| **Extrathyroidal extension** (any) | 8 (57.1%) | 27 (58.7%) | 1.066 (0.318-3.575) | 0.918 |
| microscopic | 6 (42.8%) | 20 (43.5%) | 1.026 (0.306-3.435) | 1.000 |
| macroscopic | 2 (14.3%) | 7 (15.2%) | 1.077 (0.197-5.894) | 1.000 |
| **pT category** |  |  | 1.035 (0.512-2.092) | 0.924 |
| **pN category (N1)** | 12 (85.7%) | 27 (58.7%) | 0.237 (0.047-1.182) | 0.079 |
| **M category (M1)** | 0 | 1 (2.2%) | 0.957 (0.010-92.007) | 0.985 |
| **Oncocytic changes** | 11 (78.6%) | 38 (82.6%) | 1.476 (0.854-2.551) | 0.164 |
| **Cystic changes** | 3 (21.4%) | 12 (26.1%) | 1.376 (0.360-5.266) | 0.641 |
| **BRAF^V600E^-positive** | 8 (57.1%) | n=45; 33 (73.3%) | 2.062 (0.592-7.183) | 0.255 |
| **RAS-positive** | 0 | n=45; 1 (2.2%) | 0.977 (0.010-93.834) | 0.992 |
| **Ki-67 labeling index**, (%) | 5.9 (2.4-12.1; 2.9-9.7) | n=45; 4.8 (1.0-14.3; 4.1-7.0) | 0.930 (0.772-1.120) | 0.442 |
| **p16-positive cells, tumor invasive areas** | 14 (100%) | n=45; 44 (97.8%) | 1.077 (0.603-1.921) | 0.803 |
| **PD-L1-positive TECs**^1^ (>0) | 9 (64.3%) | n=45; 21 (46.7%) | 0.868 (0.465-1.620) | 0.656 |
| **PD-L1-positive TAICs**^2^ (>0) | 11 (78.6%) | n=45; 33 (73.3%) | 0.432 (0.157-1.190) | 0.104 |
| **PD-1-positive TAICS** (>0) | 9 (64.3%) | n=45; 26 (57.8%) | 0.637 (0.234-1.731) | 0.377 |
| **Coexisting thyroid cancer** | 0 | 0 | NA^3^ | NA |
| **Concomitant nodular disease** | 0 | 14 (30.4%) | 12.397 (0.654-255.823) | 0.093 |
| **Concomitant diffuse disease** | 5 (35.7%) | 11 (23.9%) | 0.813 (0.526-1.255) | 0.349 |
| **Concomitant Graves’ disease** | 0 | 1 (2.2%) | 0.957 (0.010-92.007) | 0.985 |
| **Concomitant chronic thyroiditis** | 5 (35.7%) | 10 (21.7%) | 0.500 (0.136-1.832) | 0.295 |
| **Lymph node dissection performed** | 12 (85.7%) | 35 (76.1%) | 0.759 (0.461-1.249) | 0.278 |
| **RIT cycles** | 2 (1-4; 1-2) | 2 (1-10; 1-2) | 1.031 (0.655-1.625) | 0.894 |
| **Cumulative RI activity**, MBq | 5744 (1425-16255; 4661-8190) | 5456 (1831-54036; 2757-8432) | 1.000 (1.000-1.000) | 0.799 |

^1^ Tumor epithelial cells

^2^ Tumor-associated immune cells

^3^ Not available

Number in bold indicates statistical significance

**Supplementary Table 2** Comparison of the radiogenic and sporadic primary metastases

| **Parameter** | **Sporadic, n=12** | **Radiogenic, n=27** | **OR (95% CI)** | **p-value** |
| --- | --- | --- | --- | --- |
| **Sex F/M** (%M); (ref=F) | 10/2 (20.0%) | 21/6 (28.6%) | 1.429 (0.244-8.375) | 0.693 |
| **Age at operation**, years | 17.1 (8.7-25.3; 14.2-23.6) | 32.3 (19.6-46.4; 27.1-36.4) | 1.446 (1.120-1.868) | **0.005** |
| **Lymph nodes removed** | 10 (1-29; 8.5-17) | 10 (1-20; 5-12) | 0.926 (0.828-1.036) | 0.179 |
| **Metastatic lymph nodes** | 7 (1-15; 4.5-11) | 7 (1-17; 3-10) | 0.966 (0.829-1.126) | 0.659 |
| **Largest metastatic lymph node size**, mm | 15 (3-50; 11-20) | 20 (3-100; 7-39) | 1.031 (0.983-1.082) | 0.206 |
| **Dominant growth pattern** |  |  | 0.694 (0.331-1.456) | 0.334 |
| **Histological subtype** |  |  | 1.089 (0.732-1.622) | 0.673 |
| **Tall cell features** | 3 (25.0%) | 11 (40.7%) | 2.062 (0.453-9.388) | 0.349 |
| **Hobnail features** | 0 | 3 (11.1%) | 3.573 (0.109-117.283) | 0.475 |
| **Extranodal extension** | 2 (16.7%) | 11 (40.7%) | 2.927 (0.577-14.840) | 0.195 |
| **Oncocytic changes** | 9 (75.0%) | 22 (81.5%) | 1.467 (0.288-7.470) | 0.645 |
| **Cystic changes** | 2 (16.7%) | 12 (44.4%) | 3.386 (0.672-17.054) | 0.139 |
| **BRAF^V600E^-positive** | n=11; 5 (45.5%) | n=24; 19 (79.2%) | 4.560 (0.975-21.322) | 0.054 |
| **RAS-positive** | 0 | n=24; 1 (4.2%) | 1.469 (0.015-143.690) | 0.869 |
| **Ki-67 labeling index**, (%) | n=10; 5.8 (3.2-9.7; 4.1-7.0) | n=24; 4.3 (1.2-12.2; 3.2-7.7) | 0.975 (0.759-1.252) | 0.840 |
| **p16-positive cells, tumor invasive areas** | n=11; 10 (90.9%) | n=24; 23 (95.8%) | 2.300 (0.130-40.545) | 0.569 |
| **PD-L1-positive TECs**^1^ (>0) | n=11; 5 (45.5%) | n=24; 14 (58.3%) | 1.680 (0.399-7.075) | 0.479 |
| **PD-L1-positive TAICs**^2^ (>0) | n=11; 10 (90.9%) | n=24; 20 (83.3%) | 0.500 (0.049-5.083) | 0.558 |
| **PD-1-positive TAICs** (>0) | n=11; 10 (90.9%) | n=24; 16 (66.7%) | 0.200 (0.022-1.849) | 0.156 |
| **Coexisting thyroid cancer** | 0 | 0 | NA^3^ | NA |
| **Concomitant nodular disease** | 0 | 8 (29.6%) | 10.899 (0.486-244.410) | 0.132 |
| **Concomitant diffuse disease** | 5 (41.7%) | 7 (25.9%) | 0.490 (0.117-2.057) | 0.330 |
| **Concomitant Graves’ disease** | 0 | 0 | NA | NA |
| **Concomitant chronic thyroiditis** | 5 (41.7%) | 7 (25.9%) | 0.490 (0.117-2.057) | 0.330 |
| **RIT cycles** | 1.5 (1-4; 1-2.5) | 2 (1-10; 1-2) | 1.209 (0.696-2.100) | 0.501 |
| **Cumulative RI activity**, MBq | 4726 (1425-16255; 4548-8446) | 7080 (1831-54036; 4087-9096) | 1.000 (1.000-1.000) | 0.467 |

^1^ Tumor epithelial cells

^2^ Tumor-associated immune cells

^3^ Not available

Number in bold indicates statistical significance

**Supplementary Table 3** Comparison of the radiogenic and sporadic recurrent RAI-R metastases

| **Parameter** | **Sporadic, n=14** | **Radiogenic, n=46** | **OR (95% CI)** | **p-value** |
| --- | --- | --- | --- | --- |
| **Sex F/M** (%M); (ref=F) | 11/3 (21.4%) | 33/13 (28.3%) | 1.444 (0.346-6.029) | 0.614 |
| **Age at operation**, years | 21.0 (9.9-25.9; 17.9-24.1) | 36.1 (21.3-49.0; 30.1-41.1) | 1.592 (1.176-2.155) | **0.003** |
| **Lymph nodes removed** | 5 (1-12; 3-5) | 4 (1-22; 2-6) | 1.009 (0.870-1.170) | 0.904 |
| **Metastatic lymph nodes** | 3 (1-7; 2-4) | 2 (1-18; 1-4) | 1.017 (0.829-1.248) | 0.872 |
| **Largest metastatic lymph node size**, mm | 11 (6-25; 10-15) | 13 (6-25; 10-15) | 1.026 (0.908-1.159) | 0.684 |
| **Dominant growth pattern** |  |  | 0.864 (0.464-1.607) | 0.643 |
| **Histological subtype** |  |  | 1.250 (0.818-1.908) | 0.302 |
| **Tall cell features** | 4 (28.6%) | 22 (47.8%) | 2.143 (0.604-7.597) | 0.238 |
| **Hobnail features** | 2 (14.3%) | 8 (17.4%) | 1.104 (0.220-5.541) | 0.904 |
| **Extranodal extension** | 6 (42.9%) | 17 (37.0%) | 0.782 (0.232-2.637) | 0.691 |
| **Oncocytic changes** | 10 (71.4%) | 38 (82.6%) | 1.900 (0.474-7.611) | 0.365 |
| **Cystic changes** | 5 (35.7%) | 24 (52.2%) | 1.964 (0.570-6.764) | 0.285 |
| **BRAF^V600E^-positive** | 8 (57.1%) | n=45; 33 (73.3%) | 2.062 (0.592-7.183) | 0.255 |
| **RAS-positive** | 0 | n=45; 1 (2.2%) | 0.977 (0.010-93.834) | 0.992 |
| **Ki-67 labeling index**, (%) | n=10; 5.4 (2.5-14.6; 3.3-7.0) | n=45; 5.4 (1.6-11.1; 3.5-7.6) | 0.991 (0.790-1.242) | 0.935 |
| **p16-positive cells, tumor invasive areas** | 14 (100%) | n=45; 45 (100%) | NA^3^ | NA |
| **PD-L1-positive TECs**^1^ (>0) | 5 (35.7%) | n=45; 24 (53.3%) | 2.057 (0.595-7.110) | 0.254 |
| **PD-L1-positive TAICs**^2^ (>0) | 14 (100%) | n=45; 34 (75.6%) | 0.103 (0.005-2.123) | 0.141 |
| **PD-1-positive TAICs** (>0) | 11 (78.6%) | n=45; 26 (57.8%) | 0.373 (0.091-1.524) | 0.170 |

^1^ Tumor epithelial cells

^2^ Tumor-associated immune cells

^3^ Not available

Number in bold indicates statistical significance

**Supplementary Table 4** Statistical details of the agreement coefficients between the PT, PMTS and RMTS characteristics

| **Parameters** | **Percent agreement** | **Coefficient (95% CI)** | **p-value^1^** |
| --- | --- | --- | --- |
|  | *PT and PMTS and RMTS* | | |
| **Dominant growth pattern**^2^ | 58.3 | 0.422 (0.260-0.583) | **2.41E-06** |
| **Histological subtype**^2^ | 33.3 | 0.239 (0.134-0.343) | **2.47E-05** |
| **Tall cell features**^3^ | 72.2 | 0.456 (0.249-0.662) | **4.28E-05** |
| **Hobnail features**^3^ | 83.3 | 0.795 (0.672-0.917) | **<1.00E-06** |
| **Extrathyroidal and extranodal extension**^3^ | 63.3 | 0.279 (0.079-0.480) | **0.007** |
| **Oncocytic changes**^3^ | 81.7 | 0.729 (0.573-0.885) | **2.86E-13** |
| **Cystic changes**^3^ | 61.7 | 0.278 (0.048-0.509) | **0.019** |
| **BRAF^V600E^-positive**^4^ | 100.0 | 1.000 (0.966-1.000) | **<1.00E-06** |
| **NRAS^Q61R^-positive**^4^ | 100.0 | 1.000 (0.966-1.000) | **<1.00E-06** |
| **Ki-67 labeling index**, %^4^ | 78.9 | 0.431 (0.286-0.575) | **1.59E-07** |
| **p16-positive TEC, invasive areas**^4^ | 96.0 | 0.921 (0.824-1.000) | **<1.00E-06** |
|  | *PT and PMTS* | | |
| **Dominant growth pattern** | 59.0 | 0.436 (0.195-0.677) | **6.19E-04** |
| **Histological subtype** | 38.5 | 0.298 (0.109-0.488) | **0.0025** |
| **Tall cell features** | 76.9 | 0.549 (0.257-0.84) | **3.88E-04** |
| **Hobnail features** | 89.7 | 0.883 (0.677-1.000) | **5.91E-12** |
| **Extrathyroidal and extranodal extension** | 53.8 | 0.086 (-0.232-0.405) | **0.591** |
| **Oncocytic changes** | 92.3 | 0.885 (0.667-1.000) | **3.44E-11** |
| **Cystic changes** | 66.7 | 0.419 (0.104-0.734) | **0.010** |
| **BRAF^V600E^-positive** | 100.0 | 1.000 (0.780-1.000) | **8.14E-13** |
| **NRAS^Q61R^-positive** | 100.0 | 1.000 (0.780-1.000) | **8.14E-13** |
| **Ki-67 labeling index**, % | 77.5 | 0.391 (0.107-0.675) | **0.008** |
| **p16-positive TEC, invasive areas** | 94.2 | 0.886 (0.635-1.000) | **2.18E-09** |
|  | *PT and RMTS* | | |
| **Dominant growth pattern** | 53.3 | 0.360 (0.171-0.549) | **3.29E-04** |
| **Histological subtype** | 26.7 | 0.140 (0.003-0.276) | **0.045** |
| **Tall cell features** | 68.3 | 0.375 (0.131-0.619) | **0.003** |
| **Hobnail features** | 80.0 | 0.748 (0.592-0.904) | **1.29E-13** |
| **Extrathyroidal and extranodal extension** | 56.7 | 0.134 (-0.124-0.393) | **0.303** |
| **Oncocytic changes** | 78.3 | 0.686 (0.502-0.870) | **4.20E-10** |
| **Cystic changes** | 56.7 | 0.191 (-0.084-0.465) | **0.169** |
| **BRAF^V600E^-positive** | 100.0 | 1.000 (0.966-1.000) | **<1.00E-06** |
| **NRAS^Q61R^-positive** | 100.0 | 1.000 (0.966-1.000) | **<1.00E-06** |
| **Ki-67 labeling index**, % | 78.5 | 0.420 (0.231-0.609) | **3.90E-05** |
| **p16-positive TEC, invasive areas** | 96.3 | 0.966 (0.891-1.000) | **<1.00E-06** |
|  | *PMTS and RMTS* | | |
| **Dominant growth pattern** | 82.1 | 0.748 (0.521-0.974) | **1.29E-08** |
| **Histological subtype** | 53.8 | 0.460 (0.251-0.668) | **4.43E-05** |
| **Tall cell features** | 84.6 | 0.701 (0.433-0.968) | **2.22E-06** |
| **Hobnail features** | 84.6 | 0.800 (0.573-1.000) | **2.10E-09** |
| **Extranodal extension** | 74.4 | 0.521 (0.226-0.816) | **7.93E-04** |
| **Oncocytic changes** | 82.1 | 0.732 (0.484-0.981) | **1.94E-07** |
| **Cystic changes** | 79.5 | 0.594 (0.309-0.879) | **1.02E-04** |
| **BRAF^V600E^-positive** | 100.0 | 1.000 (0.780-1.000) | **8.14E-13** |
| **NRAS^Q61R^-positive** | 100.0 | 1.000 (0.780-1.000) | **8.14E-13** |
| **Ki-67 labeling index**, % | 79.4 | 0.444 (0.220-0.668) | **2.02E-04** |
| p16-positive TEC, invasive areas | 94.3 | 0.886 (0.635-1.000) | **2.18E-09** |

^1^ non-adjusted

^2^ Gwet's AC1 (irrCAC R package)

^3^ Gwet's weighted AC1, ordinal (irrCAC R package)

^4^ Brennan-Prediger weighted kappa, ordinal (irrCAC R package)

Numbers in bold indicate statistical significance

**Supplementary Table 5** Statistical details of the correlation coefficients between the PT, PMTS and RMTS characteristics

| **Parameters** | **Coefficient (95% CI)** | **p-value^1^** |
| --- | --- | --- |
|  | *PT and PMTS* |  |
| **Dominant growth pattern**^2^ | 0.327 (0.040-0.613) | **0.034** |
| **Histological subtype**^2^ | 0.262 (-0.035-0.558) | 0.089 |
| **Tall cell features**^3^ | 0.528 (0.260-0.795) | **0.001** |
| **Hobnail features**^3^ | 0.278 (-0.235-0.790) | 0.087 |
| **Extrathyroidal and extranodal extension**^3^ | 0.291 (0.055-0.527) | 0.073 |
| **Oncocytic changes**^3^ | 0.755 (0.521-0.989) | **3.26E-06** |
| **Cystic changes**^3^ | 0.207 (-0.117-0.531) | 0.202 |
| **BRAF^V600E^-positive**^3^ | 1.000 (1.000-1.000) | **5.51E-09** |
| **NRAS^Q61R^-positive**^3^ | 1.000 (1.000-1.000) | **5.51E-09** |
| **Ki-67 labeling index**, %^2^ | 0.137 (-0.194-0.467) | 0.415 |
| **p16-positive TEC, invasive areas**^3^ | NA^4^ | NA |
|  | *PT and RMTS* |  |
| **Dominant growth pattern** | 0.153 (-0.091-0.398) | 0.224 |
| **Histological subtype** | -0.053 (-0.317-0.212) | 0.696 |
| **Tall cell features** | 0.358 (0.121-0.596) | **0.006** |
| **Hobnail features** | 0.060 (-0.232-0.351) | 0.646 |
| **Extrathyroidal and extranodal extension** | 0.180 (-0.064-0.424) | 0.168 |
| **Oncocytic changes** | 0.302 (0.008-0.595) | **0.021** |
| **Cystic changes** | 0.135 (-0.115-0.385) | 0.301 |
| **BRAF^V600E^-positive** | 1.000 (1.000-1.000) | **5.51E-09** |
| **NRAS^Q61R^-positive** | 1.000 (1.000-1.000) | **5.51E-09** |
| **Ki-67 labeling index**, % | 0.084 (-0.170-0.338) | 0.516 |
| **p16-positive TEC, invasive areas** | NA | NA |
|  | *PMTS and RMTS* |  |
| **Dominant growth pattern** | 0.734 (0.526-0.942) | **8.20E-12** |
| **Histological subtype** | 0.305 (-0.036-0.646) | 0.081 |
| **Tall cell features** | 0.680 (0.447-0.913) | **2.78E-05** |
| **Hobnail features** | 0.367 (-0.036-0.769) | **0.024** |
| **Extranodal extension** | 0.447 (0.157-0.738) | **0.006** |
| **Oncocytic changes** | 0.424 (0.069-0.780) | **0.009** |
| **Cystic changes** | 0.594 (0.348-0.840) | **2.52E-04** |
| **BRAF^V600E^-positive** | 1.000 (1.000-1.000) | **2.62E-14** |
| **NRAS^Q61R^-positive** | 1.000 (1.000-1.000) | **2.62E-14** |
| **Ki-67 labeling index**, % | 0.113 (-0.219-0.444) | **5.07E-01** |
| **p16-positive TEC, invasive areas** | NA | NA |

^1^ non-adjusted value

^2^ Spearman's rho (SAS PROC FREQ)

^3^ Kendall's tau-b (SAS macro kendall)

^4^ not available due to zero variance

Numbers in bold indicate statistical significance

**Supplementary Table 6** Individual tumor characteristics as risk factors for recurrent RAI-R metastasis

| **Parameters** | **HR (95% CI)** | **p-value** | | |
| --- | --- | --- | --- | --- |
| **Sex F/M** (ref=F) | 1.112 (0.619-2.000) | 0.722 | | |
| **Radiation exposure** | 0.526 (0.283-0.980) | **0.043** | | |
| **Age at operation**, years | 1.023 (0.990-1.057) | 0.167 | | |
| **Age at exposure**, years (exposed only) | 1.018 (0.955-1.086) | 0.579 | | |
| **Period of latency**, years (exposed only) | 1.117 (1.047-1.192) | **0.001** | | |
| **Radiation dose to the thyroid**, mGy (exposed only) | 0.999 (0.997-1.000) | 0.141 | | |
| **Probability of causation (POC)**, %^1^ | 0.987 (0.975-0.999) | **0.036** | | |
| 0 – ≤ 25% | 1.750 (0.941-3.255) | 0.077 | | |
| > 25 – 50% | 0.637 (0.251-1.616) | 0.342 | | |
| > 50 – 75% | 0.871 (0.372-2.041) | 0.751 | | |
| > 75 – 100% | 0.344 (0.082-1.436) | 0.143 | | |
| **Probability of causation (POC)**, % (exposed only) | 0.990 (0.977-1.003) | 0.118 | | |
| **Tumor size**, mm | 0.997 (0.982-1.013) | 0.709 | | |
| microcarcinoma (≤10 mm) | 1.117 (0.497-2.513) | 0.789 | | |
| 11 – 20 mm | 1.354 (0.794-2.308) | 0.266 | | |
| 21 – 40 mm | 0.592 (0.337-1.037) | 0.067 | | |
| >40 mm | 1.473 (0.685-3.165) | 0.321 | | |
| **Dominant growth pattern** | 1.070 (0.800-1.431) | 0.648 | | |
| papillary | 0.813 (0.471-1.403) | 0.456 | | |
| follicular | 1.892 (0.736-4.860) | 0.185 | | |
| solid-trabecular | 1.039 (0.575-1.876) | 0.900 | | |
| **Histological subtype** | 0.979 (0.850-1.127) | 0.765 | | |
| papillary | 1.050 (0.571-1.932) | 0.875 | | |
| follicular | 1.276 (0.308-5.285) | 0.737 | | |
| solid-trabecular | 1.046 (0.414-2.643) | 0.925 | | |
| conventional | 0.984 (0.585-1.655) | 0.952 | | |
| rare | 0.899 (0.451-1.794) | 0.763 | | |
| **Tall cell features** | 0.793 (0.473-1.329) | 0.378 |  |  |
| **Hobnail features** | 1.106 (0.396-3.088) | 0.848 |  |  |
| **Full tumor capsule** | 1.094 (0.393-3.045) | 0.864 |  |  |
| **Multifocality** | 1.130 (0.647-1.974) | 0.668 | |  |
| **Lymphatic/vascular invasion** | 0.876 (0.486-1.580) | 0.660 | |  |
| **Extrathyroidal extension** (any) | 1.152 (0.682-1.946) | 0.596 | |  |
| microscopic | 1.100 (0.647-1.868) | 0.725 | |  |
| macroscopic | 1.103 (0.539-2.258) | 0.789 | |  |
| **pT category** | 0.995 (0.718-1.379) | 0.976 | |  |
| pT1 | 1.296 (0.772-2.176) | 0.326 | |  |
| pT1a | 1.117 (0.497-2.513) | 0.789 | |  |
| pT1b | 1.264 (0.741-2.156) | 0.389 | |  |
| pT2 | 0.487 (0.248-0.957) | **0.037** | |  |
| pT3 | 1.406 (0.784-2.521) | 0.253 | |  |
| pT3a | 1.321 (0.472-3.694) | 0.596 | |  |
| pT3b | 1.365 (0.719-2.594) | 0.342 | |  |
|  |  |  | |  |
| **pN category** (N1) | 1.422 (0.824-2.455) | 0.206 | |  |
| pN1a | 0.828 (0.464-1.477) | 0.523 | |  |
| pN1b | 1.777 (1.034-3.053) | **0.038** | |  |
| **M category** (M1) | 2.577 (0.345-19.26) | 0.356 | |  |
| **Oncocytic changes** | 1.204 (0.621-2.337) | 0.582 | | |
| focal | 1.334 (0.650-2.740) | 0.432 | | |
| moderate | 1.034 (0.611-1.748) | 0.902 | | |
| severe | 0.841 (0.452-1.567) | 0.586 | | |
| oncocytic metastasis | 1.635 (0.503-5.317) | 0.414 | | |
| **Cystic changes** | 1.039 (0.574-1.880) | 0.900 | | |
| **BRAF^V600E^-positive** | 0.732 (0.416-1.288) | 0.279 | | |
| **RAS-positive** | 0.813 (0.111-5.928) | 0.838 | | |
| **Ki-67 labeling index** | 0.986 (0.907-1.072) | 0.746 | | |
| 0 – 5% | 0.879 (0.521-1.482) | 0.628 | | |
| >5 – 10% | 1.188 (0.701-2.014) | 0.523 | | |
| >10% | 0.910 (0.389-2.133) | 0.829 | | |
| **p16-positive cells, tumor invasive areas** | 0.859 (0.664-1.112) | 0.249 | | |
| ≤ 25% | 1.365 (0.776-2.399) | 0.280 | | |
| > 25 – 50% | 1.121 (0.611-2.055) | 0.713 | | |
| > 50 – 75% | 0.669 (0.383-1.168) | 0.157 | | |
| > 75 – 100% | 1.062 (0.448-2.515) | 0.892 | | |
| **Coexisting thyroid cancer** | NA^2^ | NA | | |
| **Concomitant nodular disease** | 1.081 (0.587-1.992) | 0.802 | | |
| **Concomitant diffuse disease** | 1.258 (0.703-2.251) | 0.439 | | |
| **Concomitant Graves' disease** | 0.497 (0.068-3.620) | 0.490 | | |
| **Concomitant chronic thyroiditis** | 1.414 (0.778-2.567) | 0.255 | | |
| **Lymph node dissection performed** | 1.545 (0.827-2.888) | 0.173 | | |
| level ≥ 6 | 0.713 (0.400-1.271) | 0.251 | | |
| level 1 – 5 | 1.932 (1.141-3.272) | **0.014** | | |
| **RIT cycles** | 1.220 (1.023-1.456) | **0.027** | | |
| **Cumulative RAI activity**, MBq | 1.000 (1.000-1.000) | **0.006** | | |

^1^ POC of PTCs in non-exposed patients was assumed to be equal to 0

^2^ Not available

Numbers in bold indicate statistical significance

**Supplementary Table 7** Characteristics of the primary tumors of RAI-R recurrent PTCs by the immune checkpoint status

| **Parameters** | **ICS-positive, n=14** | **ICS-negative, n=45** | **p-value**^1^ |
| --- | --- | --- | --- |
|  | number (%) or median (range; IQR) | number (%) or median (range; IQR) |  |
| **Sex F/M** (%M, F:M ratio) | 14/0 (0%; 14:0) | 30/15 (33.3%; 2:1) | **0.013** |
| **Exposed/nonexposed** (% exposed) | 11/3 (78.6%) | 34/11 (75.6%) | 1.000 |
| **Age at operation**, years | 33.3 (8.7-43.5; 26.5-36.5) | 25.3 (12.8-48.4; 219-35.2) | 0.364 |
| **Age at exposure**, years | n=11; 11.1 (4.0-14.7; 6.1-13.1) | n=34; 8.9 (0-18.3; 2.2-11.9) | 0.354 |
| **Period of latency**, years | n=11; 23.9 (15.5-30.4; 21.8-29.2) | n=34; 22.1 (12.6-31.0; 18.6-26.7) | 0.203 |
| **Radiation dose to the thyroid**, mGy | n=11; 21.3 (3.1-801.9; 8.5-82.9) | n=34; 32.1 (2.3-825.1; 23.6-64.4) | 0.635 |
| **Probability of causation (POC),** % | n=11; 6.6 (0.8-79.5; 3.1-38.1) | n=34; 13.0 (0.8-86.5; 8.3-31.2) | 0.609 |
| ≤ 25% | 10 (71.4%) | 35 (77.8%) | 0.722 |
| > 25 – 50% | 1 (7.1%) | 5 (11.1%) | 1.000 |
| > 50 – 75% | 2 (14.3%) | 4 (8.9%) | 0.620 |
| > 75 – 100% | 1 (7.1%) | 1 (2.2%) | 0.421 |
| **Tumor size**, mm | 25.0 (11-52; 20-35) | 18 (6-105; 13-35) | 0.218 |
| < 10 mm (microcarcinoma) | 0 | 6 (13.3%) | 0.319 |
| 11 – 20 mm | 4 (28.6%) | 21 (46.7%) | 0.354 |
| 21 – 40 mm | 8 (57.1%) | 12 (26.7%) | 0.053 |
| > 40 mm | 2 (14.3%) | 6 (13.3%) | 1.000 |
| **Full tumor capsule** | 0 | 4 (8.9%) | 0.564 |
| **Dominant growth pattern** |  |  |  |
| papillary | 8 (57.1%) | 31 (68.9%) | 0.521 |
| follicular | 0 | 5 (11.1%) | 0.325 |
| solid-trabecular | 6 (42.9%) | 9 (20.0%) | 0.156 |
| **Tall cell features** | 6 (42.9%) | 21 (46.7%) | 1.000 |
| **Hobnail features** | 1 (7.1%) | 3 (6.7%) | 1.000 |
| **Histological subtype** |  |  |  |
| papillary | 2 (14.3%) | 12 (26.7%) | 0.482 |
| follicular | 0 | 2 (4.4%) | 1.000 |
| solid-trabecular | 0 | 5 (11.1%) | 0.325 |
| conventional | 7 (50.0%) | 21 (46.7%) | 1.000 |
| rare | 5 (35.7%)^2^ | 5 (11.1%)^3^ | **0.047** |
| **Oncocytic changes** | 13 (92.9%) | 36 (80.0%) | 0.425 |
| ≤ 25% focal | 2 (14.3%) | 7 (15.6%) | 0.157 |
| > 25 – 50% moderate | 3 (21.4%) | 21 (46.7%) | 0.125 |
| > 50 – 75% severe | 6 (42.9%) | 7 (15.6%) | 0.060 |
| > 75 – 100% oncocytic tumor | 2 (14.3%) | 1 (2.2%) | 0.137 |
| ≤ 50% | 5 (4.4%) | 28 (62.3%) | 0.125 |
| > 50% | 8 (57.2%) | 8 (17.8%) | **0.013** |
| **Cystic changes** | 2 (14.3%) | 13 (28.9%) | 0.483 |
| ≤ 25% focal | 2 (14.3%) | 12 (26.7%) | 0.482 |
| > 25 – 50% moderate | 0 | 1 (2.2%) | 1.000 |
| > 50 – 75% severe | 0 | 0 | NA |
| > 75 – 100% cystic tumor | 0 | 0 | NA |
| **BRAF^V600E^-positive** | 10 (71.4%) | 31 (68.9%) | 1.000 |
| **RAS-positive** | 0 | 1 (2.2%) | 1.000 |
|  |  |  |  |
| **Ki-67 labeling index**, % | 7.2 (3.8-14.2; 5.6-9.7) | 4.5 (1.0-14.3; 3.3-6.0) | **0.003** |
| 0 – 5% | 3 (21.4%) | 26 (57.8%) | **0.030** |
| > 5 – 10% | 9 (64.3%) | 15 (33.3%) | 0.061 |
| > 10% | 2 (14.3%) | 4 (8.9%) | 0.620 |
| **p16-positive TEC**^4^**, tumor invasive areas** | 14 (100%) | 44 (97.8%) | 1.000 |
| ≤ 25% | 2 (14.3%) | 16 (35.6%) | 0.189 |
| > 25 – 50% | 2 (14.3%) | 12 (26.7%) | 0.482 |
| > 50 – 75% | 6 (42.9%) | 14 (31.1%) | 0.521 |
| > 75 – 100% | 4 (28.6%) | 2 (4.4%) | **0.024** |
| ≤ 50% | 4 (28.6%) | 29 (64.4%) | **0.030** |
| > 50% | 10 (71.4%) | 16 (35.6%) | **0.030** |
| **PD-L1-positive TEC** | 14 (100%) | 16 (35.6%) | **1.70E-05** |
| ≤ 25% | 0 | 14 (31.1%) | **9.02E-12** |
| > 25 – 50% | 11 (78.6%) | 2 (4.4%) | **9.01E-08** |
| > 50 – 75% | 3 (21.4%) | 0 | **0.011** |
| > 75 – 100% | 0 | 0 | NA |
| **PD-L1-positive TAIC**^5^ | 14 (100%) | 30 (66.7%) | **0.013** |
| ≤ 25% | 8 (57.1%) | 27 (60.0%) | 1.000 |
| > 25 – 50% | 6 (42.9%) | 3 (6.7%) | 0.004 |
| > 50 – 75% | 0 | 0 | NA |
| > 75 – 100% | 0 | 0 | NA |
| **PD-1-positive TAIC** | 14 (100%) | 21 (46.7%) | **3.02E-04** |
| ≤ 25% | 11 (78.6%) | 20 (44.4%) | **0.034** |
| > 25 – 50% | 3 (21.4%) | 1 (2.2%) | **0.038** |
| > 50 – 75% | 0 | 0 | NA |
| > 75 – 100% | 0 | 0 | NA |
| **Multifocality** | 6 (42.9%) | 13 (28.9%) | 0.345 |
| **Lymphatic/vascular invasion** | 10 (71.4%) | 35 (77.8%) | 0.722 |
| **Extrathyroidal extension (any)** | 8 (57.1%) | 26 (57.8%) | 1.000 |
| microscopic | 6 (42.9%) | 19 (42.2%) | 1.000 |
| macroscopic | 2 (14.3%) | 7 (15.6%) | 1.000 |
| **pT category** |  |  |  |
| pT1 | 5 (35.7%) | 26 (57.8%) | 0.221 |
| pT1a | 0 | 6 (13.3%) | 0.319 |
| pT1b | 5 (35.7%) | 20 (44.4%) | 0.758 |
| pT2 | 6 (42.9%) | 6 (13.3%) | **0.026** |
| pT3 | 3 (21.4%) | 13 (28.9%) | 0.738 |
| pT3a | 1 (7.1%) | 3 (6.7%) | 1.000 |
| pT3b | 2 (14.3%) | 10 (22.2%) | 0.712 |
| **pN category** (N1) | 12 (85.7%) | 26 (57.8%) | 0.108 |
| pN1a | 6 (42.9%) | 8 (17.8%) | 0.075 |
| pN1b | 6 (42.9%) | 18 (40.0%) | 1.000 |
| **M category** (M1) | 0 | 1 (2.2%) | 1.000 |
| **Coexisting thyroid cancer** | 0 | 0 |  |
| **Concomitant benign nodules** | 5 (35.7%) | 9 (20.0%) | 0.285 |
| **Concomitant Graves' disease** | 0 | 1 (2.2%) | 1.000 |
| **Chronic thyroiditis** | 9 (64.3%) | 6 (13.3%) | **4.41E-04** |
| **Thyroid surgery volume** |  |  |  |
| total thyroidectomy | 14 (100%) | 45 (100%) | 1.000 |
| organ-preserving operation | 0 | 0 | NA |
|  |  |  |  |
| **Lymph node dissection performed** | 13 (92.9%) | 33 (73.3%) | 0.159 |
| level ≥ 6 | 4 (28.6%) | 11 (24.4%) | 0.738 |
| level 1 – 5 | 9 (64.3%) | 22 (48.9%) | 0.370 |
| **RIT performed** | 14 (100) | 45 (100) | 1.000 |
| **RIT cycles** | 1 (1-5; 1-2) | 2 (1-10; 1-2) | 0.401 |
| **Cumulative RI activity**, MBq | 4530 (1425-20887; 2405-8878) | 5582 (1962-54036; 4255-8175) | 0.470 |
| **Follow-up**, years | 8.7 (2.0-17.0; 5.0-14.1) | 8.2 (1.0-21.0; 4.7-13.7) | 0.845 |
| **Lymph node recurrence** (reoperated after 6 mo) | 14 (100%) | 45 (100%) | 1.000 |
| **Time to recurrence**, years | 2.3 (0.7-9.3; 1.0-3.0) | 1.5 (0.5-19.5; 0.9-3.8) | 0.418 |
| **Primary metastases** |  |  |  |
| PD-L1 positive TEC (>25.0%) | n=11; 10 (90.9%) | n=24; 2 (8.3%) | **4.00E-06** |
| PD-L1 positive TAIC (any) | 11 (100%) | 19 (79.2%) | 0.157 |
| PD-1 positive TAIC (any) | 11 (100%) | 15 (62.5%) | **0.033** |
| ICS-positive | 10 (90.9%) | 2 (8.3%) | **4.00E-06** |
| **Recurrent metastases** |  |  |  |
| PD-L1 positive TEC (>25.0%) | 13 (92.9%) | 4 (8.9%) | **7.57E-09** |
| PD-L1 positive TAIC (any) | 14 (100%) | 34 (75.6%) | 0.051 |
| PD-1 positive TAIC (any) | 14 (100%) | 23 (51.1%) | **9.05E-04** |
| ICS-positive | 13 (92.6%) | 4 (8.9%) | **7.57E-09** |

^1^Univariate analysis

^2^Three Warthin-like and two tall cell subtypes

^3^Five tall cell subtype

^4^Tumor epithelial cells

^5^Tumor-associated immune cells

Numbers in bold indicate statistical significance

**Supplementary Table 8** Characteristics of the primary metastases of RAI-R recurrent PTCs by the immune checkpoint status

| **Parameters** | **ICS-positive, n=12** | **ICS-negative, n=23** | **p-value**^1^ |
| --- | --- | --- | --- |
|  | number (%) or median (range; IQR) | number (%) or median (range; IQR) |  |
| **Sex F/M** (%M, F:M ratio) | 11/1 (8.3%; 11:1) | 17/6 (26.1%; 2.8:1) | 0.380 |
| **Exposed/nonexposed** (% exposed) | 10/2 (83.3%) | 14/9 (60.9%) | 0.259 |
| **Age at operation**, years | 35.1 (8.7-43.5; 28.4-38.0) | 24.6 (12.1-46.4; 21.0-31.2) | 0.053 |
| **Age at exposure**, years | n=10; 12.6 (4.0-17.5; 6.5-14.4) | n-14; 6.0 (0-18.1; 1.4-11.4) | 0.172 |
| **Period of latency**, years | n=10; 24.5 (18.6-30.4; 21.7-29.5) | n=14; 24.3 (12.6-29.1; 19.5-27.2) | 0.437 |
| **Radiation dose to the thyroid**, mGy | n=10; 23.8 (3.1-251.9; 8.7-63.6) | n=14; 37.6 (5.7-162.9; 23.2-84.4) | 0.285 |
| **Probability of causation (POC),** % | n=10; 6.3 (0.8-60.6; 3.7-25.2) | n=14; 18.1 (1.5-60.5; 9.2-47.9) | 0.192 |
| ≤ 25% | 7 (70.0%) | 8 (57.1%) | 0.679 |
| > 25 – 50% | 1 (100%) | 4 (28.6%) | 0.358 |
| > 50 – 75% | 2 (20.0%) | 2 (14.3%) | 1.000 |
| > 75 – 100% | 0 | 0 | NA |
| **Lymph nodes removed** | 10 (1-20; 6-15) | 10 (1-29; 7-14) | 0.771 |
| **Metastatic lymph nodes** | 7 (1-15; 4-11) | 7 (1-17; 3-10) | 0.986 |
| **Largest metastatic lymph node size**, mm | 17 (7-40; 11-27) | 17 (5-100; 12-45) | 0.520 |
| **Dominant growth pattern** |  |  |  |
| papillary | 3 (25.0%) | 13 (56.5%) | 0.152 |
| follicular | 0 | 4 (17.4%) | 0.275 |
| solid-trabecular | 9 (75.0%) | 6 (26.1%) | **0.011** |
| **Tall cell features** | 6 (50.0%) | 8 (34.8%) | 0.477 |
| **Hobnail features** | 2 (16.7) | 1 (4.3) | 0.266 |
| **Histological subtype** |  |  |  |
| papillary | 1 (8.3%) | 6 (26.1%) | 0.380 |
| follicular | 0 | 2 (8.7%) | 0.536 |
| solid-trabecular | 7 (58.3%) | 4 (17.4%) | **0.022** |
| conventional | 3 (25.0%) | 8 (34.8%) | 0.709 |
| rare | 1 (8.3%)^2^ | 3 (13.0%)^3^ | 1.000 |
| **Oncocytic changes** | 12 (100%) | 17 (73.9%) | 0.074 |
| ≤ 25% focal | 2 (16.7%) | 5 (21.7%) | 1.000 |
| > 25 – 50% moderate | 3 (25.0%) | 6 (26.1%) | 1.000 |
| > 50 – 75% severe | 3 (25.0%) | 5 (21.7%) | 1.000 |
| > 75 – 100% oncocytic metastasis | 4 (33.3%) | 1 (4.3%) | **0.038** |
| ≤ 50% | 5 (41.7%) | 11 (47.8%) | 1.000 |
| > 50% | 7 (58.3%) | 6 (26.1%) | 0.079 |
| **Cystic changes** | 2 (16.7%) | 11 (47.8%) | 0.139 |
| ≤ 25% focal | 1 (8.3%) | 6 (26.1%) | 0.380 |
| > 25 – 50% moderate | 0 | 3 (13.0%) | 0.536 |
| > 50 – 75% severe | 1 (8.3%) | 0 | 0.343 |
| > 75 – 100% cystic metastasis | 0 | 2 (8.7%) | 0.536 |
| **Extranodal extension** | 4 (33.3%) | 9 (39.1) | 1.000 |
| **BRAF-positive** | 7 (58.3%) | 17 (73.9%) | 0.451 |
| **RAS-positive** | 0 | 1 (4.3%) | 1.000 |
|  |  |  |  |
|  |  |  |  |
| **Ki-67 labeling index**, % | 6.1 (3.0-11.9; 4.2-9.9) | n=22; 4.2 (1.2-12.2; 3.2-6.3) | 0.087 |
| 0 – 5% | 5 (41.7%) | n=22; 13 (59.1%) | 0.475 |
| > 5 – 10% | 5 (41.7%) | n=22; 9 (40.9%) | 1.000 |
| > 10% | 2 (16.7%) | n=22; 0 | 0.118 |
| **p16-positive TEC^4^, invasive areas** | 12 (100%) | 21 (91.3%) | 0.536 |
| ≤ 25% | 1 (8.3%) | 6 (26.1%) | 0.380 |
| > 25 – 50% | 2 (16.7%) | 5 (21.7%) | 1.000 |
| > 50 – 75% | 1 (8.3%) | 6 (26.1%) | 0.380 |
| > 75 – 100% | 8 (66.7%) | 4 (17.4%) | **0.007** |
| ≤ 50% | 3 (25.0%) | 11 (47.8%) | 0.282 |
| > 50% | 9 (75.0%) | 10 (43.5%) | 0.282 |
| **PD-L1-positive TEC** | 12 (100%) | 7 (30.4%) | **6.30E-05** |
| ≤ 25% | 0 | 7 (30.4%) | 0.070 |
| > 25 – 50% | 6 (50.0%) | 0 | **5.69E-04** |
| > 50 – 75% | 6 (50.0%) | 0 | **5.69E-04** |
| > 75 – 100% | 0 | 0 | NA |
| **PD-L1-positive TAIC**^5^ | 12 (100%) | 18 (78.3%) | 0.141 |
| ≤ 25% | 2 (16.7%) | 15 (65.2%) | **0.012** |
| > 25 – 50% | 10 (83.3%) | 3 (13.0%) | **8.10E-05** |
| > 50 – 75% | 0 | 0 | NA |
| > 75 – 100% | 0 | 0 | NA |
| **PD-1-positive TAIC** | 12 (100%) | 14 (60.9%) | **0.015** |
| ≤ 25% | 5 (41.7%) | 11 (47.8%) | 1.000 |
| > 25 – 50% | 7 (58.3%) | 3 (13.0%) | **0.015** |
| > 50 – 75% | 0 | 0 | NA |
| > 75 – 100% | 0 | 0 | NA |
| **Primary tumors** |  |  |  |
| PD-L1 positive TEC (>25.0%) | 10 (83.3%) | 1 (4.3%) | **4.00E-06** |
| PD-L1 positive TAIC (any) | 12 (100%) | 16 (69.6%) | 0.070 |
| PD-1 positive TAIC (any) | 12 (100%) | 9 (39.1%) | **6.13E-04** |
| ICS-positive | 10 (83.3%) | 1 (4.3%) | **4.00E-06** |
| **Recurrent metastases** |  |  |  |
| PD-L1 positive TEC (>25.0%) | 12 (100%) | 1 (4.3%) | **1.56E-08** |
| PD-L1 positive TAIC (any) | 12 (100%) | 19 (82.6%) | 0.275 |
| PD-1 positive TAIC (any) | 12 (100%) | 11 (47.8%) | **0.002** |
| ICS-positive | 12 (100%) | 1 (4.3%) | **1.56E-08** |

^1^Univariate analysis

^2^One hobnail subtype

^3^Three tall cell subtype

^4^Tumor epithelial cells

^5^Tumor-associated immune cells

Numbers in bold indicate statistical significance

**Supplementary Table 9** Characteristics of the recurrent metastases of RAI-R recurrent PTCs by the immune checkpoint status

| **Parameters** | **ICS-positive, n=17** | **ICS-negative, n=42** | **p-value**^1^ |
| --- | --- | --- | --- |
|  | number (%) or median (range; IQR) | number (%) or median (range; IQR) |  |
| **Sex F/M** (%M, F:M ratio) | 16/1 (5.9%; 16:1) | 28/14 (33.3%; 2:1) | **0.045** |
| **Exposed/nonexposed** (% exposed) | 15/2 (88.2%) | 30/12 (71.4%) | 0.310 |
| **Age at operation**, years | 33.4 (8.7-43.5; 27.9-36.1) | 25.2 (12.1-48.4; 20.3-34.2) | 0.082 |
| **Age at exposure**, years | n=15; 10.4 (1.3-17.5; 4.5-13.1) | n=30; 9.5 (0-18.3; 2.2-11.9) | 0.563 |
| **Period of latency**, years | n=15; 23.9 (15.5-30.4; 21.8-28.3) | n=30; 22.0 (12.6-31.0; 18.4-26.0) | 0.149 |
| **Radiation dose to the thyroid**, mGy | n=15; 26.0 (3.1-801.9; 12.3-82.9) | n=30; 33.4 (2.3-825.1; 23.6-64.4) | 0.324 |
| **Probability of causation (POC),** % | n=15; 9.2 (0.8-79.5; 4.8-38.1) | n=30; 13.0 (0.8-86.5; 8.3-31.2) | 0.455 |
| ≤ 25% | 10 (66.7%) | 21 (70.0%) | 1.000 |
| > 25 – 50% | 1 (6.7%) | 5 (16.7%) | 0.647 |
| > 50 – 75% | 3 (20.0%) | 3 (10.0%) | 0.384 |
| > 75 – 100% | 1 (6.7%) | 1 (3.3%) | 1.000 |
| **Lymph nodes removed** | 5 (1-22; 3-8) | 4 (1-18; 2-6) | 0.292 |
| **Metastatic lymph nodes** | 3 (1-18; 2-5) | 2 (1-12; 1-4) | 0.140 |
| **Largest metastatic lymph node size**, mm | 12 (6-25; 10-15) | 13 (6-25; 10-15) | 0.767 |
| **Dominant growth pattern** |  |  |  |
| papillary | 1 (5.9%) | 27 (64.3%) | **3.10E-05** |
| follicular | 0 | 4 (9.5%) | 0.314 |
| solid-trabecular | 16 (94.1%) | 11 (26.2%) | **2.00E-06** |
| **Tall cell features** | 9 (52.9%) | 17 (40.5%) | 0.403 |
| **Hobnail features** | 3 (17.6%) | 7 (16.7%) | 1.000 |
| **Histological subtype** |  |  |  |
| papillary | 0 | 15 (35.7%) | **0.003** |
| follicular | 0 | 2 (4.8%) | 1.000 |
| solid-trabecular | 13 (76.5%) | 10 (23.8%) | **2.96E-04** |
| conventional | 3 (17.6%) | 12 (28.6%) | 0.516 |
| rare | 1 (5.9%)^2^ | 3 (7.1%)^2^ | 1.000 |
| **Oncocytic changes** | 17 (100%) | 31 (73.8%) | **0.024** |
| ≤ 25% focal | 0 | 7 (16.7%) | 0.096 |
| > 25 – 50% moderate | 3 (17.6%) | 13 (31.0%) | 0.353 |
| > 50 – 75% severe | 6 (35.3%) | 5 (11.9%) | 0.062 |
| > 75 – 100% oncocytic tumor | 8 (47.1%) | 6 (14.3%) | **0.015** |
| ≤ 50% | 3 (17.6%) | 31 (73.8%) | **1.11E-04** |
| > 50% | 14 (82.4%) | 11 (26.2%) | **1.11E-04** |
| **Cystic changes** | 5 (29.4%) | 23 (54.8) | 0.092 |
| ≤ 25% focal | 2 (11.8%) | 7 (16.7%) | 1.000 |
| > 25 – 50% moderate | 2 (11.8%) | 7 (16.7%) | 1.000 |
| > 50 – 75% severe | 0 | 2 (4.8%) |  |
| > 75 – 100% cystic metastasis | 1 (5.9%) | 7 (16.7%) | 0.417 |
| **Extranodal extension** | 7 (41.2%) | 16 (38.1%) | 1.000 |
| **BRAF-positive** | 12 (70.6%) | 29 (69.0%) | 1.000 |
| **RAS-positive** | 0 | 1 (2.4%) | 1.000 |
|  |  |  |  |
|  |  |  |  |
| **Ki-67 labeling index**, % | 7.6 (2.8-14.6; 4.0-9.7) | 5.1 (1.6-10.0; 3.5-6.2) | **0.041** |
| 0 – 5% | 6 (35.3%) | 21 (50.0%) | 0.392 |
| > 5 – 10% | 9 (52.9%) | 21 (50.0%) | 1.000 |
| > 10% | 2 (11.8%) | 0 | 0.079 |
| **p16-positive TEC**^3^**, invasive areas** | 17 (100%) | 42 (100%) | 1.000 |
| ≤ 25% | 1 (5.9%) | 11 (26.2%) | 0.150 |
| > 25 – 50% | 1 (5.9%) | 11 (26.2%) | 0.150 |
| > 50 – 75% | 4 (23.5%) | 12 (28.6%) | 0.759 |
| > 75 – 100% | 11 (64.7%) | 8 (19.0%) | **0.001** |
| ≤ 50% | 2 (11.8%) | 22 (52.4%) | **0.007** |
| > 50% | 15 (88.2%) | 20 (47.6%) | **0.007** |
| **PD-L1-positive TEC** | 17 (100%) | 12 (28.6%) | **1.87E-07** |
| ≤ 25% | 0 | 12 (28.6%) | **0.013** |
| > 25 – 50% | 7 (41.2%) | 0 | **5.70E-05** |
| > 50 – 75% | 10 (58.8%) | 0 | **3.10E-07** |
| > 75 – 100% | 0 | 0 | NA |
| **PD-L1-positive TAIC**^4^ | 17 (100%) | 31 (73.8%) | **0.024** |
| ≤ 25% | 2 (11.8%) | 26 (61.9%) | **5.07E-04** |
| > 25 – 50% | 15 (88.2%) | 5 (11.9%) | **4.21E-08** |
| > 50 – 75% | 0 | 0 | NA |
| > 75 – 100% | 0 | 0 | NA |
| **PD-1-positive TAIC** | 17 (100%) | 20 (47.6%) | **6.70E-05** |
| ≤ 25% | 9 (52.9%) | 20 (47.6%) | 0.779 |
| > 25 – 50% | 8 (47.1%) | 0 | **1.10E-05** |
| > 50 – 75% | 0 | 0 | NA |
| > 75 – 100% | 0 | 0 | NA |
| **Lymph node dissection performed** | 17 (100%) | 42 (100%) | NA |
| level ≥ 6 | 10 (58.8%) | 17 (40.5%) | 0.254 |
| level 1 – 5 | 7 (41.2%) | 25 (59.5%) | 0.254 |
| **Time to recurrence**, years | 1.8 (0.7-9.3; 0.9-2.8) | 1.4 (0.5-19.5; 0.9-4.0) | 0.610 |
| **Primary tumors** |  |  |  |
| PD-L1 positive TEC (>25.0%) | 13 (76.5%) | 3 (7.1%) | **2.54E-07** |
| PD-L1 positive TAIC (any) | 16 (94.1%) | 28 (66.7%) | **0.045** |
| PD-1 positive TAIC (any) | 16 (94.1%) | 19 (45.2%) | **4.17E-04** |
| ICS-positive | 13 (76.5%) | 1 (2.4%) | **7.57E-09** |
| **Primary metastases** |  |  |  |
| PD-L1 positive TEC (>25.0%) | n=13; 12 (92.3%) | n=22; 0 | **1.56E-08** |
| PD-L1 positive TAIC (any) | n=13; 13 (100%) | n=22; 17 (77.3%) | 0.134 |
| PD-1 positive TAIC (any) | n=13; 13 (100%) | n=22; 13 (59.1%) | **0.013** |
| ICS-positive | n=13; 12 (92.3%) | n=22; 0 | **1.56E-08** |

^1^Univariate analysis

^1^Tall cell subtype

^3^Tumor epithelial cells

^4^Tumor-associated immune cells

Numbers in bold indicate statistical significance
