# Supplementary material for: Characteristics and immune checkpoint status of radioiodine-refractory recurrent papillary thyroid carcinomas from Ukrainian Chornobyl Tissue Bank donors
Source: Front Endocrinol (Lausanne). 2024 Jan 8;14:1343848. doi: 10.3389/fendo.2023.1343848 (PMC10800488; doi:10.3389/fendo.2023.1343848)
Supplement: Supplementary file 2 [file Table_2.docx]

**Supplementary Figure 1**. Characteristics of the 60 primary tumors in the study.

**Supplementary Figure 2**. Characteristics of the 39 primary lymph node metastases in the study.

**Supplementary Figure 3**. Characteristics of the 60 RAI-R recurrent lymph node metastases in the study.

**Supplementary Figure 4**. Resemblance of clinicopathological characteristics of the PT, PMTS and RMTS.

**Supplementary Figure 5**. Correspondence analysis of clinicopathological and molecular characteristics of the PTs, PMTSs and RMTSs. Note the difference of the PTs from PMTSs and RMTSs. Results were obtained and visualized using the “ca” and “factoextra” R packages, respectively. The contribution biplot was generated using the “colgreen” option of the “map” argument of the “fviz_ca_biplot” function in the “factoextra” package.
