# Supplementary figures and images for: Characteristics and immune checkpoint status of radioiodine-refractory recurrent papillary thyroid carcinomas from Ukrainian Chornobyl Tissue Bank donors
Source: Front Endocrinol (Lausanne). 2024 Jan 8;14:1343848. doi: 10.3389/fendo.2023.1343848 (PMC10800488; doi:10.3389/fendo.2023.1343848)

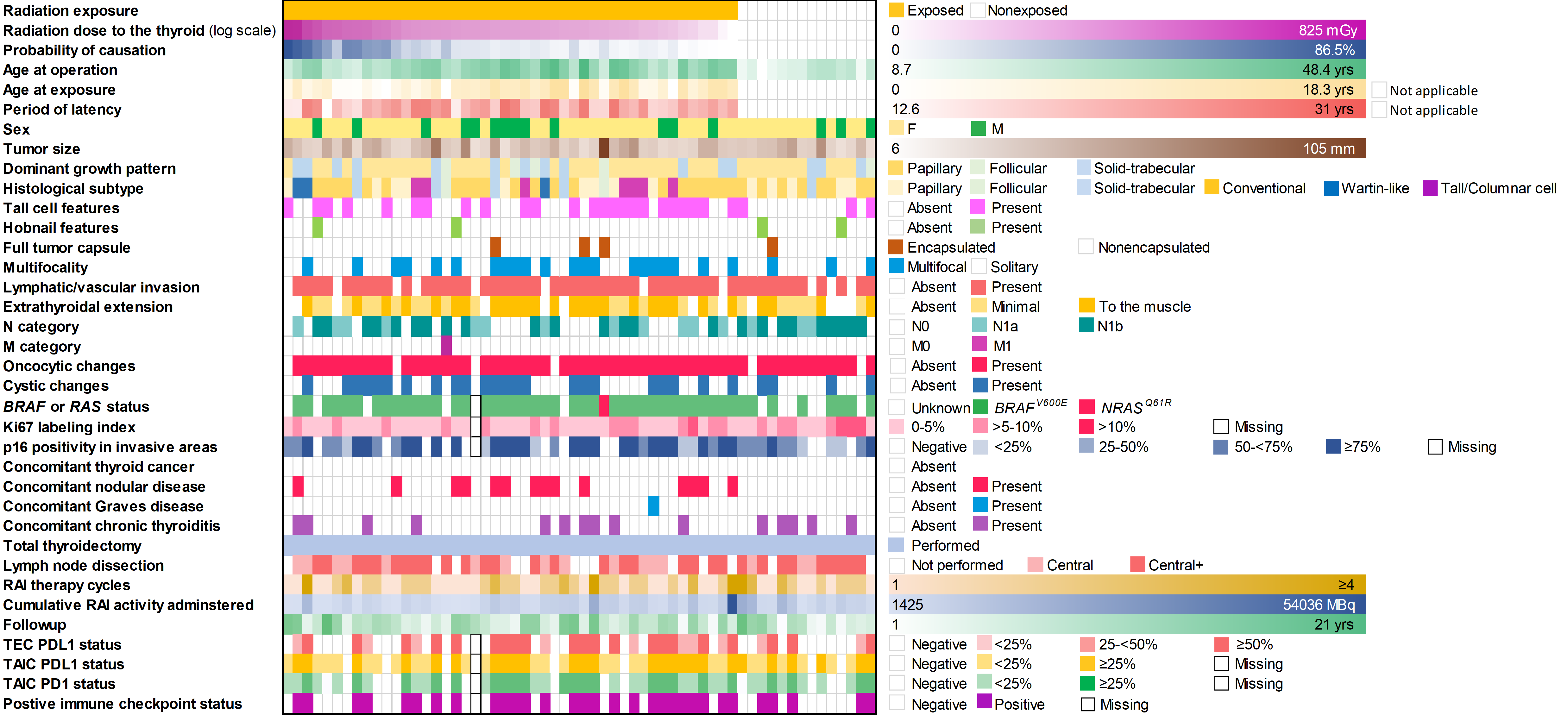

Supplement: Supplementary file 3 [file Image_1.tif]

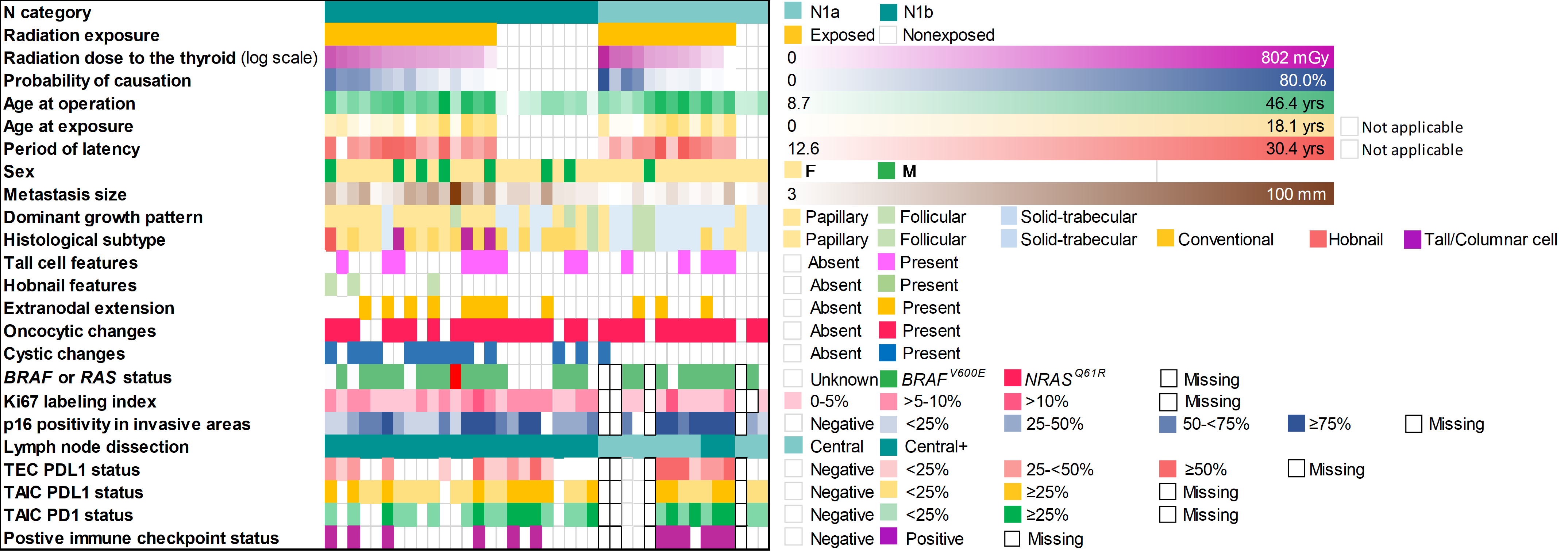

Supplement: Supplementary file 4 [file Image_2.tif]

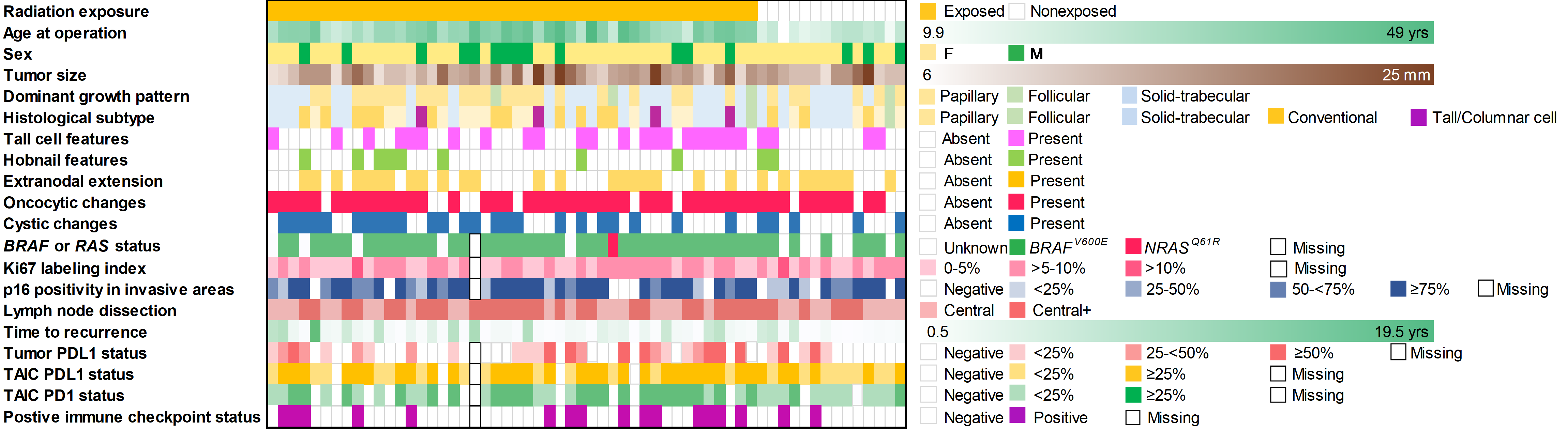

Supplement: Supplementary file 5 [file Image_3.tif]

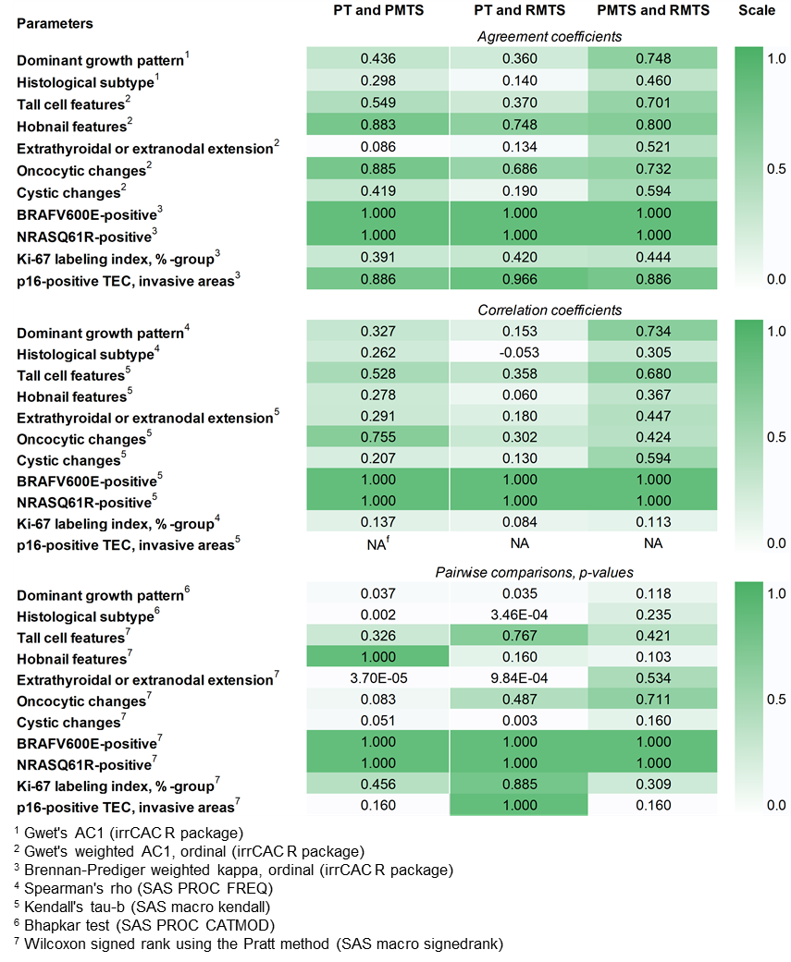

Supplement: Supplementary file 6 [file Image_4.tif]

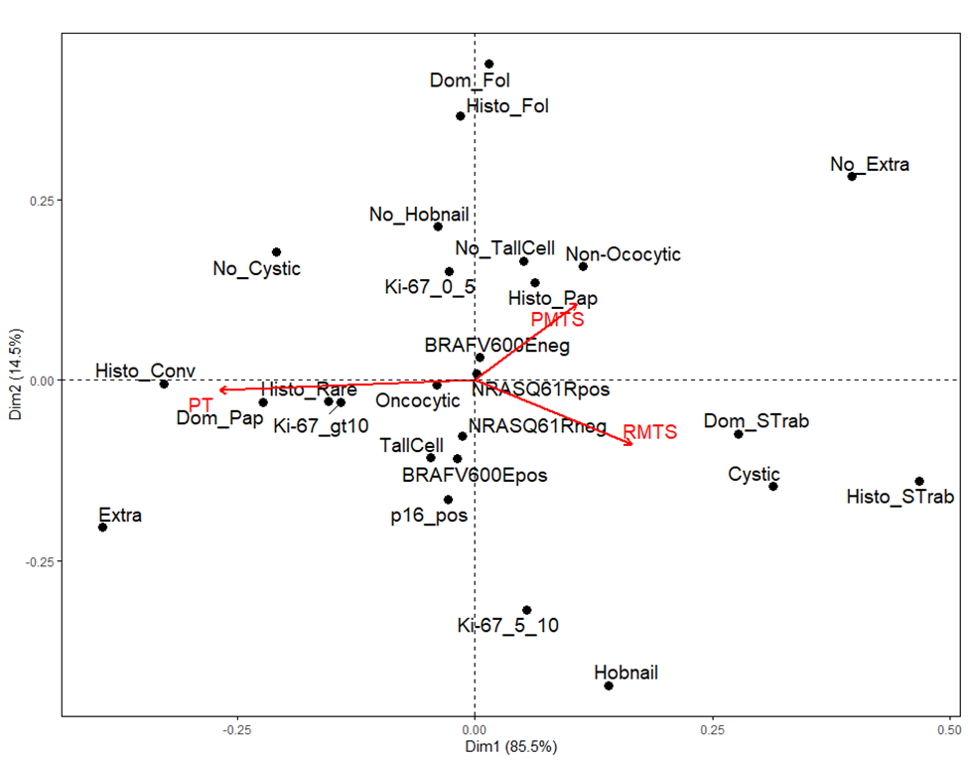

Supplement: Supplementary file 7 [file Image_5.tif]
